# Resurgence and Riemann–Hilbert Problems for Elliptic Calabi–Yau Threefolds

**DOI:** 10.1007/s00220-025-05310-9

**Published:** 2025-05-21

**Authors:** Tom Bridgeland, Iván Tulli

**Affiliations:** University of Sheffield, Sheffield, UK

## Abstract

Let *X* be a Calabi–Yau threefold with an elliptic fibration. We investigate the non-linear Riemann–Hilbert problems associated to the Donaldson–Thomas theory of fibre classes, and relate them to the Borel sum of the *A*-model topological string free energy for such classes.

## Introduction

There has been a great deal of interest recently in applying techniques from resurgence analysis to study non-perturbative effects in topological string theory. A central object in the theory is the free energy, which is a formal series in the topological string coupling $\lambda$. More precisely, the A-model closed string free energy of a Calabi–Yau (CY) threefold *X* (in the holomorphic limit) is a series of the form

1.1 $$\begin{aligned} F(\lambda ,Q)=\sum _{g\ge 0}F_g(Q)\, \lambda ^{2g-2}, \end{aligned}$$

where $F_g(Q)$ coincides with the generating function for genus *g* Gromov-Witten (GW) invariants of *X*. In studying non-perturbative effects it has been very fruitful to consider, where possible, the Borel sum of the free energy [3, 14–16, 18]. The latter depends on a choice of a ray $r\subset \mathbb {C}^*$, with the Borel sum for different choices being related by Stokes jumps. These jumps are conjecturally related to the Donaldson–Thomas (DT) invariants of *X*.

One (non-compact) CY threefold that has been much-studied from this point of view is the resolved conifold. The Borel summability of its A-model free energy was established in [3, 17, 22]. The resulting non-perturbative free energy is closely related to the log of the triple sine function $\sin _3(z\,|\,\omega _1,\omega _2,\omega _3)$^1^ [20]. The Stokes behaviour of the Borel sums was completely described in [3] and shown to be controlled by the corresponding DT invariants.

These ideas make contact with a closely related area of research which aims to use the DT invariants of a CY$_3$ category to construct a geometric structure on the space of stability conditions [6–8, 10, 11]. The geometric structure goes by the name of a Joyce structure, and is built from solutions to a collection of non-linear Riemann–Hilbert (RH) problems. These problems involve piecewise holomorphic functions on $\mathbb {C}^{*}$, with discontinuities prescribed by the DT invariants along a collection of rays, and fixed asymptotics at 0 and $\infty$. The existence and uniqueness of solutions for such problems is not known in general, but several examples have been worked out in detail [6, 9, 10].

In the case of the resolved conifold the RH problems have unique solutions [1, 9], which are closely related to the Borel sums of the free energy. More precisely, this statement holds after appropriately fixing the constant term in the asymptotics at $\epsilon =0$. The solutions are then given by functions $Y_i^{r}(\epsilon \,|\,\omega _1,\omega _2)$, with $i=1,2$, where $r=\mathbb {R}_{>0}\cdot \zeta \subset \mathbb {C}^*$ is a ray, and $\epsilon \in \mathbb {H}_r$ lies in the open half-plane $\mathbb {H}_r\subset \mathbb {C}$ centered on *r*. They can be repackaged in terms of functions $\tau ^r(\epsilon \,|\,\omega _1,\omega _2)$ satisfying the equations

1.2 $$\begin{aligned} \frac{\partial }{\partial \omega _i} \log \tau ^r(\epsilon \,|\,\omega _1,\omega _2)=\frac{1}{2\pi \textrm{i}}\cdot \frac{\partial }{\partial \epsilon } \log Y_{i}^r(\epsilon \,|\,\omega _1,\omega _2). \end{aligned}$$

It is these functions $\tau ^r(\epsilon \,|\,\omega _1,\omega _2)$ which are closely related to the Borel summation of the free energy along the ray *r* [2, 3, 9].

The goal of this paper is to address similar questions for compact CY threefolds with elliptic fibrations. We study the Borel sums of the A-model free energy and its relation to the RH problem defined by the DT invariants. We only consider the part of the free energy of *X* corresponding to fibre classes, i.e. classes $\beta \in H_2(X,\mathbb {Z})$ satisfying $\pi _*(\beta )=0$. Similarly we only consider DT invariants for coherent sheaves supported on the fibres of $\pi$.

The rest of the introduction contains a detailed summary of our main results. In general we find a similar situation to that of the resolved conifold, although our results are not as complete. One significant additional difficulty is that whereas in the case of the resolved conifold the set of Stokes directions is a closed subset of the circle, for the Borel sums considered in this paper the Stokes directions are everywhere dense. Nonetheless, we find that the free energy is again Borel summable at least along almost all non-Stokes rays, and we construct natural solutions to a weak version of the RH problem which ignores the asymptotics at $\infty$. Moreover, the two stories are again related by the equation (1.2).

### Borel sum of the free energy

In order to state our results in more detail let us briefly recall the basics of Borel summation. Consider a formal power series $F(\epsilon )=\sum _{i\ge 1} a_i \epsilon ^i$. The Borel transform is the series $f(\eta )=\sum _{i\ge 1} a_i\eta ^{i-1}/(i-1)!$. For simplicity let us assume that $f(\eta )$ is the Taylor expansion of a meromorphic function on $\mathbb {C}$ which we also denote by $f(\eta )$. We consider rays $r\subset \mathbb {C}^*$ of the form $r=\mathbb {R}_{>0}\cdot \zeta$ with $\zeta \in \mathbb {C}^*$. Such a ray is called a Stokes ray if it contains a pole of $f(\eta )$, otherwise it is a non-Stokes ray. For a given $\epsilon \in \mathbb {C}^*$ the series $F(\epsilon )$ is said to be Borel summable along a non-Stokes ray $r\subset \mathbb {C}^*$ if the integral

1.3 $$\begin{aligned} F^r(\epsilon )=\int _r e^{-\eta /\epsilon } f(\eta )\textrm{d}\eta \end{aligned}$$

exists. The Borel sum is then defined to be the value of this integral. In practice, given a non-Stokes ray $r\subset \mathbb {C}^*$, we shall only consider the Borel sum for $\epsilon$ lying in the open half-plane $\mathbb {H}_r=\{z\in \mathbb {C}:\operatorname {Re}(z/\zeta )>0\}$ centered on *r*.

We will be interested in applying Borel summation to the fibre-class free energy of the A-model topological string on an elliptic CY threefold *X*.

#### Assumption 1.1

By an elliptic CY threefold we mean a smooth projective threefold *X*, with trivial canonical bundle, equipped with a flat map $\pi :X\rightarrow B$ whose general fibre is a genus 1 curve. We always assume that *B* is smooth, that the singular fibers of $\pi$ are reduced and irreducible, and that $\pi$ has a section. We further assume that the DT/GW correspondence holds for *X*.

Under these assumptions the GW invariants of *X* in the fibre classes were computed in [21, Section B.3]. See Appendix A for a more detailed discussion. This leads to an expression

1.4 $$\begin{aligned} F_{\text {GW}}(\lambda \,|\,\tau )=-e(X)\cdot \sum _{g\ge 2} \frac{B_{2g} \, G_{2g-2}(\tau )}{4g(2g-2)} \left( \frac{\lambda }{2\pi }\right) ^{2g-2} \end{aligned}$$

for the $g\ge 2$ part of the fibre-class free energy. It is a formal series in $\lambda$ whose coefficients depend on a Kähler parameter $\tau \in \mathbb {C}$ satisfying $\operatorname {Im}(\tau )>0$. More precisely, $\tau$ is the pairing of the complexified Kähler class $B+\textrm{i}\omega \in H^2(X,\mathbb {C})$ with the fundamental class $\beta \in H_2(X,\mathbb {Z})$ of a smooth fibre of $\pi$. The expression involves the Bernoulli numbers $B_{2g}$, the Eisenstein series $G_{2g-2}(\tau )$, and the topological Euler characteristic *e*(*X*).

It will be convenient to set $2\pi \textrm{i} \epsilon =\lambda /2\pi$ and view $F_{\text {GW}}(\lambda \,|\,\tau )$ as a formal series in $\epsilon$. Furthermore, in order to relate the Borel summations of $F_{\text {GW}}(\epsilon \,|\,\tau )$ to the RH problem below, we consider instead

1.5 $$\begin{aligned} F_{\text {GW}}(\epsilon \,|\,\omega _1,\omega _2):=F_{\text {GW}}(\epsilon /\omega _1\,|\,\omega _2/\omega _1), \end{aligned}$$

where $\epsilon ,\omega _1,\omega _2\in \mathbb {C}^*$ and $\text {Im}(\omega _2/\omega _1)>0$. All our results about $F_{\text {GW}}(\epsilon \,|\,\omega _1,\omega _2)$ then specialize to results about (1.4) by simply taking $\omega _1=1$ and $\omega _2=\tau$. Our main result concerning the Borel summation of $F_{\text {GW}}(\epsilon \,|\,\omega _1,\omega _2)$ is as follows:

#### Theorem 1.2

Fix $$\omega _1,\omega _2\in \mathbb {C}^{*}$$ with $$\operatorname {Im}(\omega _2/\omega _1)>0$$.

(i) The Borel transform of $F_{\text {GW}}(\epsilon \,|\,\omega _1,\omega _2)$ is the Taylor expansion of a meromorphic function on $\mathbb {C}$ with double poles at the points $a_1\omega _1+a_2\omega _2$ with $(a_1,a_2)\in \mathbb {Z}^2\setminus \{0\}$ and no other poles.
(ii) For almost all non-Stokes rays $r\subset \mathbb {C}^*$ the series $F_{\text {GW}}(\epsilon \,|\,\omega _1,\omega _2)$ is Borel summable along *r* provided $\epsilon$ lies in the corresponding open half-plane $\mathbb {H}_r\subset \mathbb {C}^*$.

$$\square $$

More precisely, given a non-Stokes ray $r\subset \mathbb {C}^*$, there is a unique real number $\alpha \in \mathbb {R}\!\setminus \!\mathbb {Q}$ such that $\pm (\omega _1+\alpha \omega _2) \in r$. We show that the Borel sum $F^{r}_{\text {GW}}(\epsilon \,|\,\omega _1,\omega _2)$ exists and defines a holomorphic function of $\epsilon \in \mathbb {H}_r$ whenever $\alpha$ does not lie in the measure-zero subset of $\mathbb {R}\!\setminus \!\mathbb {Q}$ consisting of Liouville irrationals. For a general non-Stokes ray $r\subset \mathbb {C}^*$ we can still associate a meaningful holomorphic function of $\epsilon \in \mathbb {H}_r$ by using integrals along certain detour paths (see Sect. 2.4). These integrals reduce to the Borel sums from Theorem 1.2 whenever the ray *r* corresponds to an element $\alpha \in \mathbb {R}\!\setminus \!\mathbb {Q}$ which is not a Liouville irrational.

### DT invariants and the RH problem

Let $\pi :X\rightarrow B$ be an elliptic CY threefold satisfying Assumptions 1.1. We consider the full triangulated subcategory $\mathcal {D}(\pi )\subset D^b\operatorname {Coh}(X)$ of the bounded derived category of coherent sheaves consisting of objects whose set-theoretic support is contained in a finite union of fibres of $\pi$. The Chern character defines a homomorphism

1.6 $$\begin{aligned} \operatorname {ch}:K_0(\mathcal {D}(\pi ))\rightarrow N(\pi )\subset H^*(X,\mathbb {Z}), \end{aligned}$$

whose image $N(\pi )=\mathbb {Z}\gamma _1\oplus \mathbb {Z}\gamma _2$ is a free abelian group of rank 2. It is convenient to choose the generators $\gamma _1,\gamma _2\in N(\pi )$ so that if *E* is a rank *r*, degree *d* vector bundle supported on a smooth fibre of $\pi$ then $\operatorname {ch}(E)=-d\gamma _1+r\gamma _2$.

Given a pair of complex numbers $\omega _1,\omega _2\in \mathbb {C}^*$ with $\operatorname {Im}(\omega _2/\omega _1)>0$ there is a natural stability condition on the category $\mathcal {D}(\pi )$, uniquely defined up to the action of the even shifts, whose central charge $Z:K_0(\mathcal {D}(\pi ))\rightarrow \mathbb {C}$ is the composition of the Chern character (1.6) with the map

1.7 $$\begin{aligned} Z:N(\pi )\rightarrow \mathbb {C}, \qquad Z(a_1\gamma _1+a_2\gamma _2)=a_1\omega _1+a_2\omega _2. \end{aligned}$$

A calculation of Toda [23, Thm. 6.9] shows that the corresponding DT invariants are

1.8 $$\begin{aligned} \Omega (a_1\gamma _1+a_2\gamma _2)=-e(X), \qquad (a_1,a_2)\in \mathbb {Z}^2\setminus \{0\}. \end{aligned}$$

In [5] it was explained how to associate a RH problem to the data of the lattice $N(\pi )$, the central charge (1.7), and the DT invariants (1.8). We will recall the details of this construction in Sect. 3 below. Here we will simply state the resulting RH problem and discuss its solution. A ray $r\subset \mathbb {C}^*$ will be called a Stokes ray if it contains a point of the form $Z(\gamma )$ with $0\ne \gamma \in N(\pi )$, otherwise *r* will be called non-Stokes. As before, given a ray $r\subset \mathbb {C}^*$, we denote by $\mathbb {H}_r\subset \mathbb {C}^*$ the open half-plane centered on it.

#### Problem 1.3

For each non-Stokes ray $r\subset \mathbb {C}^*$ find holomorphic functions $Y^{r}_{i}:\mathbb {H}_r\rightarrow \mathbb {C}^*$ for $i=1,2$ such that the following statements hold.

1. If $\Delta \subset \mathbb {C}^*$ be a convex sector whose boundary consists of non-Stokes rays $r_1,r_2$ taken in clockwise order then
  1.9 $$\begin{aligned} Y^{r_2}_{i}(\epsilon )=Y^{r_1}_{i}(\epsilon )\cdot \prod _{\gamma =a_1\gamma _1+a_2\gamma _2\in Z^{-1}(\Delta )}\left( 1-e^{-Z(\gamma )/\epsilon }\right) ^{-a_i\cdot e(X)}\end{aligned}$$
  for $$\epsilon \in \mathbb {H}_{r_1}\cap \mathbb {H}_{r_2}$$ with $$0<|\epsilon |\ll 1$$.
2. As $\epsilon \rightarrow 0$ in any closed subsector of $\mathbb {H}_r$ we have $Y^r_i(\epsilon )\rightarrow 1$.
3. There is an $N>0$ such that as $\epsilon \rightarrow \infty$ in $\mathbb {H}_r$ there is a bound $|\epsilon |^{-N}< |Y^r_i(\epsilon )|<|\epsilon |^N$.

It is easy to see that if this problem has a solution then it is unique. We shall instead consider what we call the weak RH problem in which we drop condition (RH3). The resulting solution is then unique up to simultaneous multiplication of the functions $Y^r_{i}$ for all rays $r\subset \mathbb {C}^*$ by an arbitrary pair of holomorphic functions $P_i:\mathbb {C}\rightarrow \mathbb {C}^*$ satisfying $P_i(0)=1$.

In order to motivate our solution of the weak RH problem, consider again the holomorphic functions $F_{\text {GW}}^{r}(\epsilon \,|\,\omega _1,\omega _2)$ from Sect. 1.1 and define

1.10 $$\begin{aligned} \tau _{\text {GW}}^r(\epsilon \,|\,\omega _1,\omega _2):=\exp (F_{\text {GW}}^r(\epsilon \,|\,\omega _1,\omega _2)). \end{aligned}$$

As before, for a general non-Stokes ray *r* it is understood that the above expression is defined via detour paths. Furthermore, let $H_{\text {GW}}(\epsilon \,|\,\omega _1,\omega _2)$ be the formal series in $\epsilon$ without constant term satisfying

1.11 $$\begin{aligned} \frac{\partial }{\partial \epsilon }H_{\text {GW}}(\epsilon \,|\,\omega _1,\omega _2)=F_{\text {GW}}(\epsilon \,|\,\omega _1,\omega _2). \end{aligned}$$

When looking for solutions of the RH problem related to $\tau _{\text {GW}}^{r}(\epsilon \,|\,\omega _1,\omega _2)$ via (1.2), it is then natural to consider Borel summations of $2\pi \textrm{i}\cdot \frac{\partial }{\partial \omega _i} H_{\text {GW}}(\epsilon \,|\,\omega _1,\omega _2)$. Our second main result is then as follows:

#### Theorem 1.4

Fix $\omega _1,\omega _2\in \mathbb {C}^*$ with $\text {Im}(\omega _2/\omega _1)>0$. Then there exists a solution $Y^{r}_{i}(\epsilon \,|\,\omega _1,\omega _2)$ of the weak RH problem such that $\log Y_i^{r}(\epsilon \,|\,\omega _1,\omega _2)$ is the Borel sum of $2\pi \textrm{i}\frac{\partial }{\partial \omega _i} H_{\text {GW}}(\epsilon \,|\,\omega _1,\omega _2)$ along *r* for almost all non-Stokes rays *r*. Furthermore,

1.12 $$\begin{aligned} \frac{\partial }{\partial \omega _i} \log \tau ^r_{\text {GW}}(\epsilon \,|\,\omega _1,\omega _2)=\frac{1}{2\pi \textrm{i}}\cdot \frac{\partial }{\partial \epsilon } \log Y^{r}_{i}(\epsilon \,|\,\omega _1,\omega _2) \end{aligned}$$

for all non-Stokes rays *r*. $\square$

### Further remarks

Our results leave several natural challenges and questions for future research. For almost all rays $r\subset \mathbb {C}^*$ our solution to the weak RH problem can be expressed (3.14) as an integral

1.13 $$\begin{aligned} Y_{i}^r(\epsilon )=\exp \left( -\frac{e(X)}{2\pi \textrm{i}}\int _{r} \textrm{Li}_1(e^{- \eta /\epsilon })\frac{\partial }{\partial \omega _i} \mathcalligra{h}(\eta \,|\,\omega _1,\omega _2)\textrm{d}\eta \right) , \end{aligned}$$

where $\mathcalligra{h}(\eta \,|\,\omega _1,\omega _2)$ is closely related to the log of the Jacobi theta function, and is defined in terms of the Weierstrass sigma function by the equation

$$\begin{aligned} \mathcalligra{h}(u\,|\,\omega _1,\omega _2)=\log \sigma (u\,|\,\omega _1,\omega _2)-\log (u)-\tfrac{1}{2}G_2(\omega _1,\omega _2)u^2. \end{aligned}$$

An obvious challenge is to upgrade Theorem 1.4 by constructing a solution to the full Riemann–Hilbert problem. This would involve understanding the behaviour of the integral (1.13) in the limit $\epsilon \rightarrow \infty$.

In the case of the resolved conifold, the Borel sum of the free energy along a particular ray can be re-expressed [3, Theorem 2.1] in terms of the Barnes triple sine function. It is natural to ask whether the integral (1.13) can also be re-expressed in some more convenient form, and whether it can be related to known special functions.

A very interesting property of the solution to the RH problem in the case of the resolved conifold is an unexpected symmetry exchanging the parameter $\epsilon \in \mathbb {C}^*$ with the central charge parameter corresponding to the class of a point. A possible relation to S-duality in string theory was discussed in [3, Section 6]. For the RH problem considered in this paper the analogous symmetry would exchange the parammeters $\epsilon$ and $\omega _2$. Since the solutions to the RH problem already have an obvious $\operatorname {SL}_2(\mathbb {Z})$ symmetry acting on the parameters $\omega _1,\omega _2$, this perhaps hints at a possible connection with modular forms for $\operatorname {SL}_3(\mathbb {Z})$.

### Structure of the paper

In Sect. 2 we introduce some modified Weierstrass elliptic functions and summarize the results from complex analysis that we will need. In Sect. 3 we apply the contents of Sect. 2 to prove our main results, Theorems 1.2 and 1.4. The proofs of the results in Sect. 2 can be found in Sects. 4 and 5. Section 4 is concerned with the properties of the Borel transforms, whereas Sect. 5 deals with the proof of Borel summability and the existence of closely related integrals along detour paths.

## Summary of the Relevant Complex Analysis

In this section we collect the precise statements of the results from complex analysis which will be applied to prove Theorems 1.2 and 1.4 in Sect. 3. The proofs for the results in this section can be found in Sects. 4 and 5.

### Elliptic functions

Define the region

2.1 $$\begin{aligned} \mathcal {R}=\{(\omega _1,\omega _2)\in (\mathbb {C}^*)^2: \operatorname {Im}(\omega _2/\omega _1)>0\}. \end{aligned}$$

A point $(\omega _1,\omega _2)\in \mathcal {R}$ defines a lattice

2.2 $$\begin{aligned} \Lambda (\omega _1,\omega _2)=\mathbb {Z}\omega _1+\mathbb {Z}\omega _2\subset \mathbb {C}. \end{aligned}$$

We set $\Lambda ^*(\omega _1,\omega _2)=\Lambda (\omega _1,\omega _2)\setminus \{0\}$. For an even integer $n\ge 2$ we introduce the Eisenstein series

2.3 $$\begin{aligned} G_{n}(\omega _1,\omega _2)=\sum _{\omega \in \Lambda ^*(\omega _1,\omega _2)} \frac{1}{\omega ^n}=\sum _{0\ne (a_1,a_2)\in \mathbb {Z}^2} \frac{1}{(a_1\omega _1+a_2\omega _2)^n}. \end{aligned}$$

This series is absolutely convergent for $n>2$, while for $n=2$ it is only conditionally convergent. We define $G_2$ by the Eisenstein summation

2.4 $$\begin{aligned} G_2(\omega _1,\omega _2):=\sum _{a_1\in \mathbb {Z}\setminus \{0\}}\frac{1}{(a_1\omega _1)^2}+\sum _{a_2\in \mathbb {Z}\setminus \{0\}}\sum _{a_1\in \mathbb {Z}}\frac{1}{(a_1\omega _1+a_2\omega _2)^2}. \end{aligned}$$

The resulting functions $G_n(\omega _1,\omega _2)$ are holomorphic on $\mathcal {R}$ for all $n\ge 2$. These are related to the $G_{n}(\tau )$ appearing in (1.4) via $G_{n}(\tau )=G_{n}(1,\tau )$.

We recall the Weierstrass elliptic functions. The functions $\wp (u\,|\,\omega _1,\omega _2)$ and $\zeta (u\,|\,\omega _1, \omega _2)$ are meromorphic functions of $u\in \mathbb {C}$ with poles of order 2 and 1 respectively at the lattice points $\Lambda (\omega _1,\omega _2)$. The function $\sigma (u\,|\,\omega _1,\omega _2)$ is an entire function of $u\in \mathbb {C}$ with simple zeroes at the lattice points. There are relations

2.5 $$\begin{aligned} \zeta (u\,|\,\omega _1,\omega _2)=\frac{\partial }{\partial u} \log \sigma (u\,|\,\omega _1,\omega _2), \qquad \wp (u\,|\,\omega _1,\omega _2)=-\frac{\partial ^2}{\partial u^2} \log \sigma (u\,|\,\omega _1,\omega _2),\nonumber \\ \end{aligned}$$

and a Laurent expansion at $u=0$

2.6 $$\begin{aligned} \log \sigma (u\,|\,\omega _1,\omega _2)-\log (u)=-\sum _{g\ge 3} \frac{G_{2g-2}(\omega _1,\omega _2)}{2g-2} \cdot u^{2g-2}. \end{aligned}$$

We introduce minor modifications

2.7 $$\begin{aligned}  &   \mathcalligra{h}(u\,|\,\omega _1,\omega _2)=\log \sigma (u\,|\,\omega _1,\omega _2)-\log (u)-\tfrac{1}{2}G_2(\omega _1,\omega _2)u^2, \end{aligned}$$

2.8 $$\begin{aligned}  &   \tilde{\zeta }(u\,|\,\omega _1,\omega _2)= \frac{\partial }{\partial u} \mathcalligra{h}(u\,|\,\omega _1,\omega _2)=\zeta (u\,|\,\omega _1,\omega _2)-u^{-1}-G_2(\omega _1,\omega _2) \, u, \end{aligned}$$

2.9 $$\begin{aligned}  &   \tilde{\wp }(u\,|\,\omega _1,\omega _2)=-\frac{\partial ^2}{\partial u^2} \mathcalligra{h}(u\,|\,\omega _1,\omega _2)=\wp (u\,|\,\omega _1,\omega _2)-u^{-2}+G_2(\omega _1,\omega _2), \end{aligned}$$

which are holomorphic near $u=0$. Below we shall need the related functions

2.10 $$\begin{aligned}  &   \mathcalligra{f}(u\,|\,\omega _1,\omega _2)=2\tilde{\zeta }(u\,|\,\omega _1,\omega _2)-u \tilde{\wp }(u\,|\,\omega _1,\omega _2), \end{aligned}$$

2.11 $$\begin{aligned}  &   \mathcalligra{k}_i(u\,|\,\omega _1,\omega _2)=\frac{\partial }{\partial \omega _i} \mathcalligra{h} (u\,|\,\omega _1,\omega _2), \end{aligned}$$

which have poles precisely at the nonzero lattice points $\Lambda ^*(\omega _1,\omega _2)$. These are double poles in the case of $\mathcalligra{f}$ and simple poles in the case of $\mathcalligra{k}_i$. Later we will need the parity properties

2.12 $$\begin{aligned} \mathcalligra{f}(-u\,|\,\omega _1,\omega _2)=-\mathcalligra{f}(u\,|\,\omega _1,\omega _2),\qquad \mathcalligra{k}_i(-u\,|\,\omega _1,\omega _2)=\mathcalligra{k}_i(u\,|\,\omega _1,\omega _2) \end{aligned}$$

which follow immediately from the expansion (2.6).

### Borel transforms

Our starting point is the following formal power series in $\epsilon$

2.13 $$\begin{aligned} H(\epsilon \,|\,\omega _1,\omega _2)=\sum _{g\ge 2} \frac{B_{2g} \,G_{2g-2}(\omega _1,\omega _2)\, (2\pi \textrm{i})^{2g}\, \epsilon ^{2g-1}}{4g(2g-1)(2g-2)}. \end{aligned}$$

The coefficients are holomorphic functions of $(\omega _1,\omega _2)\in \mathcal {R}$ involving the Bernoulli numbers $B_{2g}$ and the Eisenstein series (2.3). We then consider the formal power series

2.14 $$\begin{aligned} F(\epsilon \,|\,\omega _1,\omega _2)=\frac{\partial }{\partial \epsilon } H(\epsilon \,|\,\omega _1,\omega _2),\qquad K_i(\epsilon \,|\,\omega _1,\omega _2)=\frac{\partial }{\partial \omega _i} H(\epsilon \,|\,\omega _1,\omega _2). \end{aligned}$$

We denote by $f(\eta \,|\,\omega _1,\omega _2)$ and $k_i(\eta \,|\,\omega _1,\omega _2)$ the Borel transforms of these series. They are power series in $\eta$ with coefficients which are holomorphic functions of $(\omega _1,\omega _2)\in \mathcal {R}$.

Note that $F(\epsilon \,|\,\omega _1,\omega _2)$ is related to the previous $F_{\text {GW}}(\epsilon \,|\,\omega _1,\omega _2)$ from (1.5) by

2.15 $$\begin{aligned} F_{\text {GW}}(\epsilon \,|\,\omega _1,\omega _2)=-\frac{e(X)}{(2\pi \textrm{i})^2}\cdot F(\epsilon \,|\,\omega _1, \omega _2). \end{aligned}$$

In particular, for $F_{\text {GW}}(\lambda \,|\,\tau )$ given in (1.4)

2.16 $$\begin{aligned} F_{\text {GW}}(\lambda \,|\,\tau )=-\frac{e(X)}{(2\pi \textrm{i})^2}\cdot F(\epsilon \,|\,1, \tau ), \qquad 2\pi \textrm{i}\epsilon =\frac{\lambda }{2\pi }. \end{aligned}$$

We choose to work with $F(\epsilon \,|\,\omega _1,\omega _2)$ rather than directly with $F_{\text {GW}}(\lambda \,|\,\tau )$ for two reasons. On the one hand, the change of variables from $\lambda$ to $\epsilon$ and the rescaling by $-e(X)/(2\pi \textrm{i})^2$ eliminates certain awkward factors from the Borel sums and the positions of the poles of the Borel transform. On the other hand, the introduction of the variables $\omega _1,\omega _2$ facilitates the relation with the RH problem.

Let us fix a point $(\omega _1,\omega _2)\in \mathcal {R}$. The following result is proved in Sect. 4.

#### Proposition 2.1

(i) The Borel transforms $f(\eta \,|\,\omega _1,\omega _2)$ and $k_i(\eta \,|\,\omega _1,\omega _2)$ have positive radius of convergence and hence define holomorphic functions in a neighbourhood of $\eta =0$.
(ii) These functions extend to meromorphic functions of $\eta \in \mathbb {C}$ with poles precisely at the nonzero lattice points $\Lambda ^*(\omega _1,\omega _2)$. The poles are double poles in the case of *f* and simple poles in the case of $k_i$.
(iii) There are explicit expressions
  2.17 $$\begin{aligned} f(\eta \,|\,\omega _1,\omega _2)= \sum _{m\ge 1} \frac{1}{m^3} \, \mathcalligra{f}\Big (\frac{\eta }{m}\,|\,\omega _1,\omega _2\Big ), \end{aligned}$$
  2.18 $$\begin{aligned} k_i(\eta \,|\,\omega _1,\omega _2)= \sum _{m\ge 1} \frac{1}{m^2} \, \mathcalligra{k}_i\Big (\frac{\eta }{m}\,|\,\omega _1,\omega _2\Big ), \end{aligned}$$
  which converge absolutely and uniformly for $\eta$ in compact subsets of $\mathbb {C}$.

$$\square $$

The Borel transform of $H(\epsilon \,|\,\omega _1,\omega _2)$ also has positive radius of convergence, but the holomorphic continuation of the resulting function $h(\eta \,|\,\omega _1,\omega _2)$ is more complicated due to the presence of logarithmic singularities, and we will not directly consider this function here.

### Irrationality measure

To define the Borel sum of the series (2.14) we must consider a Laplace-type integral of the form (1.3). Note that a ray $r\subset \mathbb {C}^*$ is non-Stokes precisely if it contains no points of the lattice $\Lambda (\omega _1,\omega _2)\subset \mathbb {C}$. Since a non-Stokes ray still comes arbitrarily close to points of $\Lambda (\omega _1,\omega _2)$, when trying to control the growth of such integrals we encounter some basic notions from Diophantine approximation which we now recall.

The irrationality measure $\mu (\alpha )$ of a real number $\alpha \in \mathbb {R}$ [12, Definition E.1] is defined to be the infimum $\mu (\alpha )=\inf R(\alpha )$ of the set

2.19 $$\begin{aligned} R(\alpha )=\big \{d \in \mathbb {R}_{>0} \; | \; 0<\left| \alpha - p/q\right| <1/q^d \text { for at most finitely many }p,q\in \mathbb {Z}, q>0\big \}.\nonumber \\ \end{aligned}$$

If $R(\alpha )= \emptyset$ we set $\mu (\alpha )=\infty$. In this case $\alpha$ is known as a Liouville irrational. We will use the following well-known properties of $\mu (\alpha )$.

#### Theorem 2.2

(i) if $$\alpha \in \mathbb {Q}$$ then $$\mu (\alpha )=1$$,
(ii) if $\alpha \in \mathbb {R}\setminus \mathbb {Q}$ then $\mu (\alpha )\ge 2$,
(iii) if $\alpha \in \mathbb {R}\setminus \mathbb {Q}$ then
  2.20 $$\begin{aligned} \mu \bigg (\frac{a\alpha +b}{c\alpha +d}\bigg )=\mu (\alpha )\text { for all } \begin{pmatrix}a& \quad b\\ c& \quad d\end{pmatrix} \in \operatorname {GL}_2(\mathbb {Z}), \end{aligned}$$
(iv) the subset $\{\alpha \in \mathbb {R}:\mu (\alpha )>2\}$ has measure zero.

#### Proof

If $\alpha \in \mathbb {Q}$ then it is easy to check that $\mu (\alpha )\ge 1$, while $\mu (\alpha )\le 1$ follows from Liouville’s theorem, which states that algebraic numbers of degree *n* satisfy $\mu (\alpha )\le n$. Part (ii) follows immediately from the Dirichlet approximation theorem, while (iv) is a Theorem due to Khinchin [19], whose proof is essentially an application of the Borel-Cantelli Lemma. We could not find a direct reference for part (iii) so we include a proof in Appendix B. $\square$

Given a point $(\omega _1,\omega _2)\in \mathcal {R}$ we can define the irrationality measure $\mu (r)\in [1,\infty ]$ of a ray $r\subset \mathbb {C}^*$ as follows. If $\pm \omega _2\in r$ we define $\mu (r)=1$. Otherwise there is a unique $\alpha \in \mathbb {R}$ such that $\pm (\omega _1+\alpha \cdot \omega _2)\in r$ and we define $\mu (r)=\mu (\alpha )$. Part (iii) of Theorem 2.2 ensures that the resulting notion depends only on the lattice $\Lambda (\omega _1,\omega _2)\subset \mathbb {C}$ rather than the specific generators $\omega _1,\omega _2$. Part (i) shows that $\mu (r)=1$ precisely if *r* contains a lattice point, and part (iv) that almost all rays have $\mu (r)=2$.

### Borel sums and integrals along detour paths

Let us again fix a point $(\omega _1,\omega _2)\in \mathcal {R}$. Recall that a ray $r\subset \mathbb {C}^*$ is non-Stokes precisely if it contains no lattice points. The following results about Borel summation are proved in Sect. 5.2.

#### Theorem 2.3

Let $r\subset \mathbb {C}^*$ be a non-Stokes ray with $\mu (r)<\infty$ and take $\epsilon \in \mathbb {H}_r$.

(i) The integrals
  2.21 $$\begin{aligned} F^r(\epsilon \,|\,\omega _1,\omega _2)=\int _r e^{-\eta /\epsilon } f(\eta \,|\,\omega _1,\omega _2)\textrm{d}\eta , \qquad K_i^r(\epsilon )=\int _r\ e^{-\eta /\epsilon } k_i(\eta \,|\,\omega _1,\omega _2)\textrm{d}\eta ,\nonumber \\ \end{aligned}$$
  are absolutely convergent and depend holomorphically on $\epsilon \in \mathbb {H}_r$. In particular, the series $F(\epsilon \,|\,\omega _1,\omega _2)$ and $K_i(\epsilon \,|\,\omega _1,\omega _2)$ are Borel summable along the ray *r*.
(ii) The Borel sums can be re-expressed as absolutely convergent integrals
  2.22 $$\begin{aligned}  &   F^r(\epsilon \,|\,\omega _1,\omega _2)=\int _{r} \textrm{Li}_2(e^{- \eta /\epsilon }) \, \mathcalligra{f}(\eta \,|\,\omega _1,\omega _2)\textrm{d}\eta , \end{aligned}$$
  2.23 $$\begin{aligned}  &   K_{i}^r(\epsilon \,|\,\omega _1,\omega _2)=\int _{r} \operatorname {Li}_1(e^{- \eta /\epsilon })\, \mathcalligra{k}_i (\eta \,|\,\omega _1,\omega _2)\textrm{d}\eta , \end{aligned}$$
  where $\textrm{Li}_k(z)$ denotes the *k*-th polylogarithm.

Note that equation (2.22) follows from (2.17) and the following formal rearrangements, which are justified in the proof of Theorem 2.3:

2.24 $$\begin{aligned} \begin{aligned} \int _r e^{-\eta /\epsilon } \, \sum _{m\ge 1} \frac{1}{m^3} \, \mathcalligra{f}\Big (\frac{\eta }{ m}\Big ) \, \textrm{d}\eta&=\sum _{m\ge 1}\, \int _r e^{-\eta /\epsilon } \, \frac{1}{m^3}\, \mathcalligra{f}\Big (\frac{\eta }{ m}\Big ) \, \textrm{d}\eta \\&=\sum _{m\ge 1}\, \int _{r} \frac{1}{m^2} \, e^{-m\eta /\epsilon }\mathcalligra{f}(\eta )\, \textrm{d}\eta \\&=\int _{r} \, \sum _{m\ge 1} \frac{1}{m^2} \, e^{-m\eta /\epsilon } \mathcalligra{f}(\eta )\, \textrm{d}\eta \\&=\int _{r} \operatorname {Li}_2(e^{-\eta /\epsilon }) \mathcalligra{f}(\eta )\, \textrm{d}\eta . \end{aligned} \end{aligned}$$

Similar remarks apply to (2.23).

**Fig. 1:**
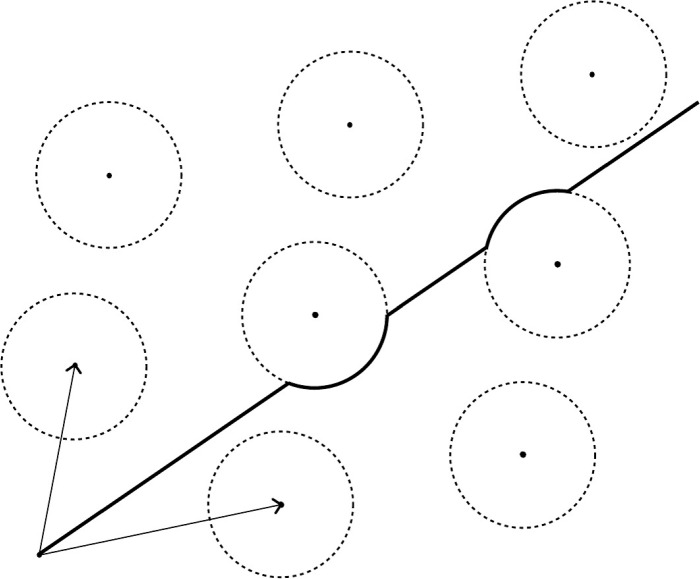
The vectors denote the generators $\omega _1$ and $\omega _2$ of the lattice $\Lambda (\omega _1,\omega _2)$. The discs are centered at the points in $\Lambda (\omega _1,\omega _2)^*$ and have radius $\delta >0$ small enough that they do not intersect. The bold path $r(\delta )$ is determined by the direction of the non-Stokes ray *r*, and takes a detour along the boundary of any disc intersected by *r*. These detours traverse arcs of the boundary of angle $<\pi$

Consider now an arbitrary non-Stokes ray $r\subset \mathbb {C}^*$. For any $0<\delta \ll \text {min}\{|\omega _1|,|\omega _2|\}$ there is a uniquely-defined detour path $r(\delta )$ which combines the ray *r* with arcs of angle $<\pi$ taken from discs of radius $\delta$ centered on points of $\Lambda ^*(\omega _1,\omega _2)$ (see Fig. 1). The following proposition is proved in Sect. 5.3.

#### Proposition 2.4

Let $r\subset \mathbb {C}^*$ be a non-Stokes ray with respect to the lattice $\Lambda (\omega _1,\omega _2)$. Then there is $D>0$ such that for all $0<\delta <D$ the integrals

2.25 $$\begin{aligned}  &   F^{r(\delta )}(\epsilon \,|\,\omega _1,\omega _2)=\int _{r(\delta )} \textrm{Li}_2(e^{- \eta /\epsilon }) \, \mathcalligra{f}(\eta \,|\,\omega _1,\omega _2)\textrm{d}\eta , \end{aligned}$$

2.26 $$\begin{aligned}  &   K_{i}^{r(\delta )}(\epsilon \,|\,\omega _1,\omega _2)=\int _{r(\delta )} \operatorname {Li}_1(e^{- \eta /\epsilon })\, \mathcalligra{k}_i (\eta \,|\,\omega _1,\omega _2)\textrm{d}\eta , \end{aligned}$$

are absolutely convergent for all $\epsilon \in \mathbb {H}_r$. The resulting integrals depend holomorphically on $\epsilon \in \mathbb {H}_{r}$, and are independent of $\delta$. Moreover

2.27 $$\begin{aligned} F^{r(\delta )}(\epsilon \,|\,\omega _1,\omega _2)=F^{r}(\epsilon \,|\,\omega _1,\omega _2), \qquad K_{i}^{r(\delta )}(\epsilon \,|\,\omega _1,\omega _2)=K_{i}^{r}(\epsilon \,|\,\omega _1,\omega _2) \end{aligned}$$

whenever $$\mu (r)<\infty $$. $$\square $$

Thus in the case of a non-Stokes ray $r\subset \mathbb {C}^*$ satisfying $\mu (r)=\infty$ we can use (2.25) and (2.26) to define substitutes for the functions (2.21), although these are no longer directly related to the Borel sums of the series $F(\epsilon \,|\,\omega _1,\omega _2)$ and $K_i(\epsilon \,|\,\omega _1,\omega _2)$.

Finally, we record how the integrals corresponding to different rays are related to each other. This proposition is proved at the end of Sect. 5.4.

#### Proposition 2.5

Let $\Delta \subset \mathbb {C}^*$ be a convex sector whose boundary consists of two rays $r_1$ and $r_2$ taken in clockwise order. Assume the rays $r_1, r_2$ are non-Stokes with respect to the lattice $\Lambda (\omega _1,\omega _2)$. Then for $\epsilon \in \mathbb {H}_{r_1}\cap \mathbb {H}_{r_2}$ and small enough $\delta >0$ we have

2.28 $$\begin{aligned} \begin{aligned} K_{i}^{r_2(\delta )}(\epsilon \,|\,\omega _1,\omega _2)-K_{i}^{r_1(\delta )}(\epsilon \,|\,\omega _1,\omega _2)&=2\pi \textrm{i}\sum _{\omega \in \Delta \cap \Lambda ^*(\omega _1,\omega _2)}a_i\cdot \log (1-e^{-\omega /\epsilon })\\ F^{r_2(\delta )}(\epsilon ,\omega _1,\omega _2)-F^{r_1(\delta )}(\epsilon ,\omega _1,\omega _2)&=2\pi \textrm{i}\sum _{\omega \in \Delta \cap \Lambda ^*(\omega _1,\omega _2)}\frac{\partial }{\partial \epsilon }\left( \epsilon \, \textrm{Li}_2(e^{-\omega /\epsilon })\right) , \end{aligned} \end{aligned}$$

where we write $\omega =a_1\omega _1+a_2\omega _2$. $\square$

## Free Energy, DT Invariants and the RH Problem

In this section we use the analytic results of the previous section to prove our main results. As in the introduction we consider a smooth projective CY threefold with an elliptic fibration $\pi :X \rightarrow B$ satisfying Assumptions 1.1.

### Free energy and its Borel sums

Recall from (1.4) that the $g\ge 2$ part of the GW generating function in the fibre classes is given by the formal power series in $\lambda$

3.1 $$\begin{aligned} F_{\text {GW}}(\lambda \,|\,\tau )=-e(X)\cdot \sum _{g\ge 2} \frac{B_{2g} \, G_{2g-2}(\tau )}{4g(2g-2)}\cdot \left( \frac{\lambda }{2\pi }\right) ^{2g-2}, \end{aligned}$$

whose coefficients depend on a Kähler parameter $\tau \in \mathbb {C}$ satisfying $\operatorname {Im}(\tau )>0$. As before, we set $2\pi \textrm{i}\epsilon =\lambda /2\pi$, and via the change of variables (1.5) consider $F_{\text {GW}}(\epsilon \,|\,\omega _1,\omega _2)$ as a function of $\epsilon \in \mathbb {C}^*$ and $(\omega _1,\omega _2)$ lying in the region

3.2 $$\begin{aligned} \mathcal {R}=\{(\omega _1,\omega _2)\in (\mathbb {C}^*)^2: \operatorname {Im}(\omega _2/\omega _1)>0\}. \end{aligned}$$

Recall from (2.15) that $F_{\text {GW}}(\epsilon \,|\,\omega _1,\omega _2)$ and $F(\epsilon \,|\,\omega _1,\omega _2)$ are related by a rescaling by $-e(X)/(2\pi \textrm{i})^2$. The following result then follows immediately by combining Proposition 2.1 and Theorem 2.3.

#### Theorem 3.1

Fix $$(\omega _1,\omega _2)\in \mathcal {R}$$.

(i) The Borel transform of the series $F_{\text {GW}}(\epsilon \,|\,\omega _1,\omega _2)$ is a meromorphic function on $\mathbb {C}$ with double poles at the non-zero lattice points $\Lambda ^*(\omega _1,\omega _2)$ and no other poles.
(ii) Suppose a non-Stokes ray $r\subset \mathbb {C}^*$ satisfies $\mu (r)<\infty$ with respect to the lattice $\Lambda (\omega _1,\omega _2)$. Then the Borel sum $F^r_{\text {GW}}(\epsilon \,|\,\omega _1,\omega _2)$ exists for all $\epsilon \in \mathbb {H}_r$.

$$\square $$

Theorem 3.1 together with part (iv) of Theorem 2.2 implies that for almost all non-Stokes rays $r\subset \mathbb {C}^*$ the Borel sum of $F_{\text {GW}}(\epsilon \,|\,\omega _1,\omega _2)$ exists for $\epsilon \in \mathbb {H}_r$. Combining Proposition 2.5 with (2.15) gives the following result relating the Borel sums along different rays.

#### Proposition 3.2

Fix $(\omega _1,\omega _2)\in \mathcal {R}$. Let $\Delta \subset \mathbb {C}^*$ be a convex sector whose boundary consists of two rays $r_1$ and $r_2$ taken in clockwise order. Assume the rays $r_1, r_2$ are non-Stokes and satisfy $\mu (r_i)<\infty$ with respect to the lattice $\Lambda (\omega _1,\omega _2)$. Then

3.3 $$\begin{aligned} F^{r_2}_{\text {GW}}(\epsilon \,|\,\omega _1,\omega _2)-F^{r_1}_{\text {GW}}(\epsilon \,|\,\omega _1,\omega _2)=-\frac{e(X)}{2\pi \textrm{i}}\cdot \sum _{\omega \in \Delta \cap \Lambda ^*(\omega _1,\omega _2)}\frac{\partial }{\partial \epsilon }\left( \epsilon \, \textrm{Li}_2(e^{-\omega /\epsilon })\right) \, \nonumber \\ \end{aligned}$$

for all $$\epsilon \in \mathbb {H}_{r_1}\cap \mathbb {H}_{r_2}$$. $$\square $$

This matches previous results on the Stokes jumps of the Borel sum of free energies and their relation to DT invariants. See for example [3, Equation 4.55] or [18, Equation 1.1 and 1.4].

### Stability conditions and DT invariants

We consider the full triangulated subcategory $\mathcal {D}(\pi )\subset D^b\operatorname {Coh}(X)$ of the bounded derived category of coherent sheaves consisting of objects whose set-theoretic support is contained in a finite union of fibres of $\pi$. The Chern characters of such objects can be viewed as elements

3.4 $$\begin{aligned} \operatorname {ch}(E)=(\operatorname {ch}_2(E),\operatorname {ch}_3(E))\in N(\pi )=N_1(\pi )\oplus N_0(X), \end{aligned}$$

where $N_1(\pi )\subset N_1(X)$ consists of curve classes contracted by $\pi$. The group $N_0(X)$ is freely generated by the class of a point, which it is convenient to denote by $-\gamma _1$. The assumption that $\pi$ has integral fibres implies that $N_1(\pi )$ is freely generated by the class $\gamma _2$ of a fibre. Then

3.5 $$\begin{aligned} \operatorname {ch}:K_0(\mathcal {D}(\pi ))\rightarrow N(\pi )=\mathbb {Z}\gamma _1\oplus \mathbb {Z}\gamma _2, \end{aligned}$$

sends a rank *r*, degree *d* bundle supported on a smooth fibre of $\pi$ to the class $-d\gamma _1+r\gamma _2$. The Riemann-Roch theorem shows that for any objects $A,B\in \mathcal {D}(\pi )$ we have

$$\begin{aligned}\chi (A,B):=\sum _{i\in \mathbb {Z}} \operatorname {dim}_{\mathbb {C}} \operatorname {Hom}_X(A,B[i])=0.\end{aligned}$$

Thus the Euler form for the category $\mathcal {D}(\pi )$ is identically zero, and we therefore also equip the group $N(\pi )$ with the zero form $\langle -,-\rangle =0$.

The definition of the subcategory $\mathcal {D}(\pi )$ ensures that the standard t-structure on $\mathcal {D}^b\operatorname {Coh}(X)$ induces a t-structure on $\mathcal {D}(\pi )$. The heart $\mathcal {A}(\pi )\subset \mathcal {D}(\pi )$ consists of coherent sheaves on *X* whose set-theoretic support is contained in a finite union of fibres of $\pi$. Fix an element $\tau \in \mathbb {C}$ with $\operatorname {Im}(\tau )>0$. Then, as in [23, Example 2.3 (iii)], there is a unique stability condition on the category $\mathcal {D}(\pi )$ whose heart is the subcategory $\mathcal {A}(\pi )\subset \mathcal {D}(\pi )$, and whose central charge $Z:K_0(\mathcal {D}(\pi ))\rightarrow \mathbb {C}$ is the composition of the Chern character (3.5) with the map

$$\begin{aligned}Z:N(\pi )\rightarrow \mathbb {C}, \qquad Z(a_1\gamma _1+a_2\gamma _2)=a_1+a_2\tau .\end{aligned}$$

There is a standard action of the group $\mathbb {C}$ on the space of stability conditions which rotates the central charge and shifts the phases of the semistable objects. Applying this to the stability conditions constructed above we obtain for each point $(\omega _1,\omega _2)\in \mathcal {R}$, a stability condition, unique up to the action of even shifts, whose central charge is the composition of (3.5) with the map

3.6 $$\begin{aligned} Z:N(\pi )\rightarrow \mathbb {C}, \qquad Z(a_1\gamma _1+a_2\gamma _2)=a_1\omega _1+a_2\omega _2. \end{aligned}$$

Since rotating stability conditions does not effect the subcategories of semistable objects, the calculation of Toda [23, Theorem 6.9] shows that the DT invariants for any of these stability conditions are given by

3.7 $$\begin{aligned} \Omega (a_1\gamma _1+a_2\gamma _2)=-e(X), \qquad (a_1,a_2)\in \mathbb {Z}^2\setminus \{0\}, \end{aligned}$$

where *e*(*X*) is the topological Euler characteristic of the complex projective variety *X*.

### Riemann–Hilbert problem

Fix a point $(\omega _1,\omega _2)\in \mathcal {R}$. The data introduced in the previous section defines what is called a BPS structure in [5]. Namely we have a finite-rank free abelian group $N(\pi )$ equipped with a skew-symmetric form $\langle -,-\rangle$, a group homomorphism $Z:N(\pi )\rightarrow \mathbb {C}$, and a map of sets $\Omega :N(\pi )\rightarrow \mathbb {Z}$ which encodes the DT invariants. Following [5], and exactly as in [9], we now explain the steps to go from this data to a RH problem.

Since the skew-symmetric form $\langle -,-\rangle$ on $N(\pi )$ is identically zero, in order to obtain a non-trivial RH problem we must first perform the doubling procedure of [5, Section 2.8]. To do this we introduce the dual abelian group $N(\pi )^{\vee }=\operatorname {Hom}_{\mathbb {Z}}(N(\pi ),\mathbb {Z})$ and consider the lattice $\Gamma =N(\pi )\oplus N(\pi )^{\vee }$ equipped with the canonical skew-symmetric pairing

3.8 $$\begin{aligned} \langle -,-\rangle :\Gamma \times \Gamma \rightarrow \mathbb {Z}, \qquad \langle (\gamma ,\lambda ),(\gamma ',\lambda ')\rangle =\lambda (\gamma ')-\lambda '(\gamma ). \end{aligned}$$

We denote by $\gamma ^\vee _i$ the basis element of $N(\pi )^{\vee }$ dual to $\gamma _i$. Thus $\langle \gamma ^\vee _i,\gamma _j\rangle =\delta _{ij}$. We extend the central charge map $Z:N(\pi )\rightarrow \mathbb {C}$ defined by (3.6) arbitrarily to a homomorphism $Z:\Gamma \rightarrow \mathbb {C}$. The choice of this extension will play no significant role below. We also extend the map of sets $\Omega :N(\pi )\rightarrow \mathbb {Z}$ to $\Gamma$ by insisting that $\Omega (\gamma )=0$ unless $\gamma \in N(\pi )\subset \Gamma$.

The resulting doubled BPS structure $(\Gamma ,Z,\Omega )$ has several special properties identified in [5]. It is convergent because for large enough $R> 0$

3.9 $$\begin{aligned} \sum _{(a_1,a_2)\in \mathbb {Z}^2\setminus \{0\}} \exp ({-R|a_1\omega _1+a_2\omega _2|})\, <\infty . \end{aligned}$$

It is moreover uncoupled since $\{\gamma \in \Gamma : \Omega (\gamma )\ne 0\}\subset N(\pi )$ and $\langle \gamma _1,\gamma _2\rangle =0$ for $\gamma _1,\gamma _2\in N(\pi )$. We can then formulate a RH problem exactly as in [9]. As well as the BPS structure $(\Gamma ,Z,\Omega )$ it depends on an element $\xi$ of the twisted torus

3.10 $$\begin{aligned} \{\xi :\Gamma \rightarrow \mathbb {C}^*:\xi (\gamma _1+\gamma _2)=(-1)^{\langle \gamma _1,\gamma _2\rangle }\xi (\gamma _1) \xi (\gamma _2)\} \end{aligned}$$

called the constant term.

Recall from Sect. 1.2 that a ray $r\subset \mathbb {C}^*$ is called a Stokes ray if it contains a point of $\Lambda (\omega _1,\omega _2)$, and otherwise a non-Stokes ray. The RH problem involves holomorphic functions $X_{\gamma }^{r}:\mathbb {H}_r\rightarrow \mathbb {C}^*$ for each non-Stokes ray *r* and each class $\gamma \in \Gamma$, where as before $\mathbb {H}_r\subset \mathbb {C}^*$ denotes the half-plane centered on *r*. Arguing as in [5, Section 5.1] we can use the fact that $(\Gamma ,Z,\Omega )$ is an uncoupled BPS structure to write for each $i=1,2$

3.11 $$\begin{aligned} X_{\gamma _i}^{r}(\epsilon )=\exp (-Z(\gamma _i)/\epsilon )\cdot \xi (\gamma _i), \qquad X_{\gamma ^\vee _i}^{r}=\exp (-Z(\gamma ^\vee _i)/\epsilon )\cdot \xi (\gamma ^\vee _i)\cdot Y_{i}^{r}(\epsilon ), \nonumber \\ \end{aligned}$$

with $Y_{i}^{r}:\mathbb {H}_r\rightarrow \mathbb {C}^*$ holomorphic. For $i=1,2$ we define

3.12 $$\begin{aligned} \ell _i:\Lambda (\omega _1,\omega _2)\rightarrow \mathbb {Z}, \qquad \ell _i(a_1\omega _1+a_2\omega _2)=a_i. \end{aligned}$$

Choose a constant term $\xi :\Gamma \rightarrow \mathbb {C}^*$ satisfying $\xi (\gamma _i)=1$ for $i=1,2$. Then the RH problem can be formulated as follows:

#### Problem 3.3

For each non-Stokes ray $r\subset \mathbb {C}^*$ find holomorphic functions $Y_{i}^{r}:\mathbb {H}_r\rightarrow \mathbb {C}^*$ with $i=1,2$ such that the following statements hold.

1. If $\Delta \subset \mathbb {C}^*$ is a convex sector whose boundary consists of non-Stokes rays $r_1,r_2$ taken in clockwise order then
  3.13 $$\begin{aligned} Y_{i}^{r_2}(\epsilon )=Y_{i}^{r_1}(\epsilon )\cdot \prod _{\omega \in \Delta (r_1,r_2)\cap \Lambda ^*}\left( 1-e^{- \omega /\epsilon }\right) ^{-\ell _i(\omega )\cdot e(X)}, \end{aligned}$$
  for $$\epsilon \in \mathbb {H}_{r_1}\cap \mathbb {H}_{r_2}$$ with $$0<|\epsilon |\ll 1$$.
2. As $\epsilon \rightarrow 0$ in any closed subsector of $\mathbb {H}_r$ we have $Y^r(\epsilon )\rightarrow 1$.
3. There is an $N>0$ such that as $\epsilon \rightarrow \infty$ in $\mathbb {H}_r$ there is a bound $|\epsilon |^{-N}< |Y^r(\epsilon )|<|\epsilon |^N$.

If this problem has a solution then it is unique [9]. We shall instead consider what we call the weak RH problem in which we drop condition (RH3). The resulting solutions are unique up to multiplication of $Y_{i}^{r}$ by arbitrary holomorphic functions $P_i:\mathbb {C}\rightarrow \mathbb {C}^*$ satisfying $P_i(0)=1$.

### Solution to the weak RH problem

We again fix a point $(\omega _1,\omega _2)\in \mathcal {R}$. Recall the functions $K_i^{r(\delta )}(\epsilon \,|\,\omega _1,\omega _2)$ defined in Proposition 2.4. For each non-Stokes ray $r\subset \mathbb {C}^*$ we define a function $Y_{i}^{r}:\mathbb {H}_r\rightarrow \mathbb {C}^*$ by

3.14 $$\begin{aligned} \begin{aligned} Y_{i}^r(\epsilon )&:=\exp \Big ( -\frac{e(X)}{2\pi \textrm{i}} \cdot K_{i}^{r(\delta )}(\epsilon \,|\,\omega _1,\omega _2)\Big )\\&=\exp \left( -\frac{e(X)}{2\pi \textrm{i}}\int _{r(\delta )} \textrm{Li}_1(e^{- \eta /\epsilon })\,\mathcalligra{k}_i(\eta \,|\,\omega _1,\omega _2)\textrm{d}\eta \right) . \end{aligned} \end{aligned}$$

The integral is absolutely convergent, holomorphic in $\epsilon \in \mathbb {H}_{r}$, and does not depend on $0< \delta \ll 1$ by Proposition 2.4. As before, we remark that for almost all non-Stokes rays *r* we have $\mu (r)<\infty$, and for such rays it follows from Proposition 2.4 and Theorem 2.3 that

3.15 $$\begin{aligned} Y_{i}^r(\epsilon )=\exp \left( -\frac{e(X)}{2\pi \textrm{i}}\int _r e^{-\eta /\epsilon }\, k_i(\eta \,|\,\omega _1,\omega _2)\textrm{d}\eta \right) . \end{aligned}$$

#### Theorem 3.4

The functions $Y_{i}^r:\mathbb {H}_r\rightarrow \mathbb {C}^*$ give a solution to the weak RH problem.

#### Proof

Let $\Delta \subset \mathbb {C}^*$ be a convex sector whose boundary consists of non-Stokes rays $r_1,r_2$ taken in clockwise order. By Proposition 2.5 it follows that for $\epsilon \in \mathbb {H}_{r_1}\cap \mathbb {H}_{r_2}$

3.16 $$\begin{aligned} \begin{aligned} Y_{i}^{r_2}(\epsilon )&=Y_{i}^{r_1}(\epsilon )\cdot \exp \left( -e(X)\sum _{\omega \in \Delta \cap \Lambda ^*}\ell _i(\omega )\cdot \log (1-e^{-\omega /\epsilon })\right) \\&=Y_{i}^{r_1}(\epsilon )\prod _{\omega \in \Delta \cap \Lambda ^*}(1-e^{-\omega /\epsilon })^{-e(X)\cdot \ell _i(\omega )} \end{aligned} \end{aligned}$$

so property (RH1) holds. Property (RH2) follows from the following lemma. $\square$

#### Lemma 3.5

Fix a non-Stokes ray $r\subset \mathbb {C}^*$ and a closed subsector $S_r\subset \mathbb {H}_r$. Then

3.17 $$\begin{aligned} \lim _{\epsilon \rightarrow 0, \;\epsilon \in S_r}K^{r(\delta )}_i(\epsilon \,|\,\omega _1,\omega _2)=0. \end{aligned}$$

#### Proof

Given the closed sector $S_r$, we can assume that $\delta >0$ is small enough such that

3.18 $$\begin{aligned} \text {Re}(\eta /\epsilon )>0 \text { for all } \eta \in r(\delta ) \text { and all } \epsilon \in S_r . \end{aligned}$$

More precisely, given $K>0$ there exists a constant $C>0$ such that

3.19 $$\begin{aligned} \text {Re}(\eta /\epsilon )=\left| \frac{\eta }{\epsilon }\right| \cos (\arg (\eta /\epsilon ))>C\cdot \left| \frac{\eta }{\epsilon }\right|>\frac{C}{K}\cdot |\eta |>0 \end{aligned}$$

for all $\eta \in r(\delta )$ and $\epsilon \in S_r$ with $|\epsilon |<K$. This in particular implies (using that $|\log (1-z)|<-\log (1-|z|)$ for $|z|<1|$) that in the same range of parameters

3.20 $$\begin{aligned} |\mathrm {Li_1}(e^{-\eta /\epsilon })\, \mathcalligra{k}_i(\eta )|<-\log (1-|e^{-\eta /\epsilon }|)\, |\mathcalligra{k}_i(\eta )|<-\log (1-e^{-C|\eta |/K})\, |\mathcalligra{k}_i(\eta )|. \nonumber \\ \end{aligned}$$

Finally, by the same argument as in Proposition 5.4 one can show that

3.21 $$\begin{aligned} -\int _{r(\delta )}\log (1-e^{-C|\eta |/K})|\mathcalligra{k}_i(\eta )||\textrm{d}\eta |<\infty , \end{aligned}$$

so by applying the dominated convergence theorem, we can interchange limits and integrals and obtain

3.22 $$\begin{aligned} \begin{aligned} \lim _{\epsilon \rightarrow 0, \;\epsilon \in S_r}K_{i}^{r(\delta )}&=\int _{r(\delta )}\textrm{Li}_1(0)\mathcalligra{k}_i(\eta ,\omega _1,\omega _2)\textrm{d}\eta =0. \end{aligned} \end{aligned}$$

$$\square $$

Fix a non-Stokes ray $r\subset \mathbb {C}^*$. For $\epsilon \in \mathbb {H}_r$ we define

3.23 $$\begin{aligned} \tau ^r_{\text {GW}}(\epsilon \,|\,\omega _1,\omega _2):=\exp \Big (-\frac{e(X)}{(2\pi \textrm{i})^2}\cdot F^{r(\delta )}(\epsilon \,|\,\omega _1, \omega _2)\Big ), \end{aligned}$$

where $0<\delta \ll 1$ and $F^{r(\delta )}(\epsilon \,|\,\omega _1, \omega _2)$ is as in Proposition 2.4. Note that by (2.15)

3.24 $$\begin{aligned} \tau ^r_{\text {GW}}(\epsilon \,|\,\omega _1, \omega _2)=\exp \Big (F^{r(\delta )}_{\text {GW}}(\epsilon \,|\,\omega _1,\omega _2)\Big ). \end{aligned}$$

In the case that $\mu (r)<\infty$ the following result relates the Borel sum of the free energy to the solution to the weak RH problem constructed above.

#### Theorem 3.6

For each $i=1,2$ there is a relation

3.25 $$\begin{aligned} \frac{\partial }{\partial \omega _i}\log \tau ^r_{\text {GW}}(\epsilon \,|\,\omega _1,\omega _2)=\frac{1}{2\pi \textrm{i}}\cdot \frac{\partial }{\partial \epsilon }\log Y_{i}^{r}(\epsilon \,|\,\omega _1,\omega _2). \end{aligned}$$

#### Proof

Using (3.23) and (3.15), showing that (3.25) holds reduces to showing that

3.26 $$\begin{aligned} \frac{\partial }{\partial \omega _i}F^{r(\delta )}(\epsilon \,|\,\omega _1,\omega _2)=\frac{\partial }{\partial \epsilon } K_i^{r(\delta )}(\epsilon \,|\,\omega _1,\omega _2). \end{aligned}$$

This easily follows by differentiating under the integral sign and integrating by parts twice using (2.8), (2.9), (2.10), and (2.11). $\square$

## Borel Transforms

In this section we prove Proposition 2.1 concerning the Borel transforms of our series. We fix a point $(\omega _1,\omega _2)\in \mathcal {R}$ throughout.

### Hadamard product

Consider again the power series

4.1 $$\begin{aligned} H(\epsilon )=\sum _{g\ge 2} \frac{B_{2g} \,G_{2g-2}\, (2\pi \textrm{i})^{2g}\epsilon ^{2g-1}}{4g(2g-1)(2g-2)} \in \epsilon \mathbb {C}[[\epsilon ]]. \end{aligned}$$

from (2.13). We have suppressed the dependence on $\omega _1,\omega _2$ from the notation. The Borel transform is the series

4.2 $$\begin{aligned} h(\eta )=\sum _{g\ge 2} \frac{B_{2g} \, G_{2g-2}\, (2\pi \textrm{i})^{2g}\eta ^{2g-2}}{2(2g-2)(2g)!}\in \mathbb {C}[[\eta ]]. \end{aligned}$$

Following the approach of [3, Section 3.1] we can write $h(\eta )$ as a Hadamard product of series $h_1(\eta )$ and $h_2(\eta )$, where

4.3 $$\begin{aligned} h_1(\eta )=-\sum _{g\ge 2} \frac{B_{2g} \, (2\pi \textrm{i})^{2g}\eta ^{2g-2}}{2 (2g)!}, \qquad h_2(\eta )=-\sum _{g\ge 2} \frac{G_{2g-2}\, \eta ^{2g-2}}{2g-2}. \end{aligned}$$

Using the defining generating series for the Bernoulli numbers we find that $h_1(\eta )$ is the Taylor expansion at the origin of a meromorphic function on $\mathbb {C}$ with poles only at the points $m\in \mathbb {Z}{\setminus }\{0\}$. Indeed

4.4 $$\begin{aligned} h_1(\eta )=(2\pi \textrm{i})^2\left( \frac{1}{2(2\pi \textrm{i}\eta )(1-e^{2\pi \textrm{i}\eta })}+\frac{1}{2(2\pi \textrm{i}\eta )^2} -\frac{1}{4(2\pi \textrm{i}\eta )} +\frac{1}{24}\right) . \end{aligned}$$

Moreover $h_2(\eta )$ is the Taylor expansion at $\eta =0$ of the function

4.5 $$\begin{aligned} \mathcalligra{h}(\eta )=\log \sigma (\eta )-\log (\eta )-\tfrac{1}{2}G_2 \eta ^2 \end{aligned}$$

introduced in Sect. 2.1. It follows from [3, Lemma 3.2] that $h(\eta )$ is the Taylor expansion at $\eta =0$ of the function $h(\eta )$ given by the anti-clockwise contour integral

4.6 $$\begin{aligned} h(\eta )=\frac{1}{2\pi \textrm{i}} \int _{ |s|=\tfrac{1}{2}} h_1(s)\, h_2(\eta /s) \, \frac{\textrm{d}s}{s}. \end{aligned}$$

This expression is valid providing $|\eta |<\tfrac{1}{2} |\omega |$ for all nonzero lattice points $\omega \in \Lambda ^*(\omega _1,\omega _2)$. This ensures that the singularities of $h_2(\eta /s)$ all lie inside the contour $|s|=\tfrac{1}{2}$. Note that the poles of $h_1(s)$ always lie outside this contour.

### Borel transforms

Recall the formal power series $F(\epsilon )$ and $K_i(\epsilon )$ defined by (2.14). Reall also the functions

4.7 $$\begin{aligned}  &   \mathcalligra{f}(\eta \,|\,\omega _1,\omega _2)=2\tilde{\zeta }(\eta \,|\,\omega _1,\omega _2)-\eta \tilde{\wp }(\eta \,|\,\omega _1,\omega _2), \end{aligned}$$

4.8 $$\begin{aligned}  &   \mathcalligra{k}_i(\eta \,|\,\omega _1,\omega _2)=\frac{\partial }{\partial \omega _i} \mathcalligra{h} (\eta \,|\,\omega _1,\omega _2). \end{aligned}$$

defined in Sect. 2.1. They are holomorphic in a neighbourhood of $\eta =0$.

#### Proposition 4.1

The Borel transforms of the series $F(\epsilon )$ and $K_i(\epsilon )$ are the Taylor expansions of holomorphic functions $f(\eta )$ and $k_i(\eta )$ defined near $\eta =0$ by the expressions

4.9 $$\begin{aligned} f(\eta )=\frac{1}{2\pi \textrm{i}} \int _{ |s|=\tfrac{1}{2}} h_1(s)\, \mathcalligra{f}(\eta /s) \, \frac{\textrm{d}s}{s^2}, \qquad k_i(\eta )=\frac{1}{2\pi \textrm{i}} \int _{ |s|=\tfrac{1}{2}} h_1(s)\, \mathcalligra{k}_i(\eta /s) \, \frac{\textrm{d}s}{s}. \nonumber \\ \end{aligned}$$

#### Proof

The expression for $k_i(\eta )$ follows immediately by differentiating (4.6) under the integral with respect to $\omega _i$. To obtain the expression for $f(\eta )$ note first that if formal series $F(\epsilon ), H(\epsilon )\in \epsilon \mathbb {C}[[\epsilon ]]$ have Borel transforms $f(\eta ), h(\eta )\in \mathbb {C}[[\eta ]]$ respectively, then

4.10 $$\begin{aligned} F(\epsilon )=\frac{\textrm{d}}{\textrm{d}\epsilon } H(\epsilon ) \implies f(\eta )=\frac{1}{\eta } \frac{\textrm{d}}{\textrm{d}\eta } \bigg (\eta ^2 \,\frac{\textrm{d}}{\textrm{d}\eta }h(\eta )\bigg ). \end{aligned}$$

Indeed, it is enough to check the case $H(\epsilon )=\epsilon ^n$ when the relation becomes

4.11 $$\begin{aligned} \frac{n\,\eta ^{n-2}}{(n-2)!}=\frac{1}{\eta } \,\frac{\textrm{d}}{\textrm{d}\eta } \bigg (\eta ^2 \frac{\textrm{d}}{\textrm{d}\eta }\bigg (\frac{\eta ^{n-1}}{(n-1)!}\bigg )\bigg ). \end{aligned}$$

Next note that (2.7) - (2.9) give

4.12 $$\begin{aligned} \frac{1}{\eta } \frac{\textrm{d}}{\textrm{d}\eta } \bigg (\eta ^2 \,\frac{\textrm{d}}{\textrm{d}\eta }\mathcalligra{h}\left( \frac{\eta }{s}\right) \bigg )=\frac{1}{s}\left( 2\tilde{\zeta }\left( \frac{\eta }{s}\right) -\frac{\eta }{s}\tilde{\wp }\left( \frac{\eta }{s}\right) \right) =\frac{1}{s}\mathcalligra{f}\left( \frac{\eta }{s}\right) . \end{aligned}$$

The result then follows by differentiating (4.6) under the integral with respect to $\eta$. $\square$

### Explicit expressions

The following result completes the proof of Proposition 2.1.

#### Proposition 4.2

The functions $f(\eta )$ and $k_i(\eta )$ extend to meromorphic functions of $\eta \in \mathbb {C}$ with poles precisely at the nonzero lattice points $\Lambda ^*(\omega _1,\omega _2)$. These poles are double poles in the case of *f* and simple poles in the case of $k_i$. There are explicit expressions

4.13 $$\begin{aligned} f(\eta )=\sum _{m\ge 1} \frac{1}{m^3} \cdot \mathcalligra{f}\Big (\frac{\eta }{m}\Big ), \qquad k_i(\eta )=\sum _{m\ge 1} \frac{1}{m^2} \cdot \mathcalligra{k}_i\Big (\frac{\eta }{ m}\Big ), \end{aligned}$$

where the two series converge uniformly and absolutely in $\eta$ on compact subsets of $\mathbb {C}$.

#### Proof

We show the result for *f*, since a similar argument applies to $k_i$. For each integer $N>0$ we consider the square contour

4.14 $$\begin{aligned} C_{N}=\big \{s\in \mathbb {C}: \max \big (|\text {Re}(s)|,|\text {Im}(s)|\big )=N+\tfrac{1}{2}\big \}, \end{aligned}$$

taken with the anti-clockwise orientation. Take $\eta \in \mathbb {C}$ such that $|\eta |<\tfrac{1}{2}|\omega |$ for all nonzero lattice points $\omega \in \Lambda ^*(\omega _1,\omega _2)$.

Note that the function $h_1(s)$ has a simple pole at each point $m\in \mathbb {Z}\setminus \{0\}$ with residue $-1/2m$. Moving the contour in (4.9) and using $\mathcalligra{f}(-\eta )=-\mathcalligra{f}(\eta )$ therefore shows that for any integer $N>0$

4.15 $$\begin{aligned} f(\eta )-\frac{1}{2\pi \textrm{i}} \int _{C_{N}} h_1(s)\, \mathcalligra{f}(\eta /s) \, \frac{\textrm{d}s}{s^2}=\sum _{m=1}^N \frac{1}{m^3} \cdot \mathcalligra{f}\Big (\frac{\eta }{m}\Big ). \end{aligned}$$

On the other hand, one easily checks using the power expansion of $\mathcalligra{f}(\eta )$ at $\eta =0$ that

4.16 $$\begin{aligned} \mathcalligra{f}(\eta /s)=\mathcal {O}(1/s), \quad \text {as } |s|\rightarrow \infty , \end{aligned}$$

while

4.17 $$\begin{aligned} \left| \frac{1}{2(2\pi \textrm{i} s)(1-e^{2\pi \textrm{i}s})}\right| <\frac{C}{N+\tfrac{1}{2}}, \quad \text {for } s\in C_N, \end{aligned}$$

for some $C>0$ independent of *N*. It then follows from (4.4) that there is some constant $D>0$ such that for all *N* sufficiently large

4.18 $$\begin{aligned} \left| \,\int _{C_{N}} h_1(s)\, \mathcalligra{f}(\eta /s) \, \frac{\textrm{d}s}{s^2}\,\right| <\frac{D}{(N+\tfrac{1}{2})^{2}}. \end{aligned}$$

Thus the integral on the left-hand side of (4.15) tends to 0 as $N\rightarrow \infty$, and (4.13) holds.

It remains to show that the series (4.13) defines a meromorphic function on $\mathbb {C}$ with double poles exactly at the points of $\Lambda ^*(\omega _1,\omega _2)$. Note that $\mathcalligra{f}(\eta )$ has poles only at the points $\Lambda ^*(\omega _1,\omega _2)$ and in particular is holomorphic at $\eta =0$.

Let $D\subset \mathbb {C}$ be small disc centered at 0 such that $D\cap \Lambda ^*(\omega _1,\omega _2)=\emptyset$ and $K\subset \mathbb {C}$ a compact subset. For $\eta \in K$ and $M>0$ sufficiently large we have $\eta /m\in D$ for $m\ge M$. In particular, the functions $\mathcalligra{f}(\eta /m)$ are holomorphic and uniformly bounded for $m\ge M$ and $\eta \in K$. It follows that the tail of the first sum in (4.13) (i.e. the sum for $m\ge M$) converges uniformly and absolutely on *K*, and hence to a holomorphic function on *K*. Since any lattice point $\omega \in \Lambda ^*(\omega _1,\omega _2)$ is a double pole of the function $\mathcalligra{f}(\eta /m)$ for a finite but non-empty set of positive integers *m*, the resulting $f(\eta )$ is a meromorphic function with double poles at $\Lambda ^*(\omega _1,\omega _2)$. $\square$

## Borel Summability and Detour Integrals

In this section we collect the results needed to show that the series $K_i(\epsilon \,|\,\omega _1,\omega _2)$ and $F(\epsilon \,|\,\omega _1,\omega _2)$ are Borel summable along almost all non-Stokes rays $r\subset \mathbb {C}^*$. More precisely, the Borel sum exists for non-Stokes rays *r* that, in the sense of Sect. 2.4, have finite irrationality measure $\mu (r)<\infty$ with respect to the lattice $\Lambda (\omega _1,\omega _2)$. For a general non-Stokes ray *r*, we define integrals along certain detour paths $r(\delta )$ which coincide with the Borel sums when $\mu (r)<\infty$.

### Key lemmas

We begin with the following useful lemmas:

#### Lemma 5.1

Fix $(\omega _1,\omega _2)\in \mathcal {R}$ and take $\omega =a_1\omega _1+a_2\omega _2\in \Lambda (\omega _1,\omega _2)$. Then the functions $\wp (\eta \,|\,\omega _1,\omega _2)$, $\zeta (\eta \,|\,\omega _1,\omega _2)$ and $\rho _i(\eta \,|\,\omega _1,\omega _2):=\partial _{\omega _i}\log (\sigma (\eta \,|\,\omega _1,\omega _2))$ satisfy

5.1 $$\begin{aligned} \begin{aligned} \wp (\eta +\omega \,|\,\omega _1,\omega _2)&=\wp (\eta \,|\,\omega _1,\omega _2),\\ \zeta (\eta +\omega \,|\,\omega _1,\omega _2)&=\zeta (\eta \,|\,\omega _1,\omega _2)+2(a_1\eta _1+a_2\eta _2), \quad \text {where} \quad \eta _i=\zeta (\omega _i/2\,|\,\omega _1,\omega _2),\\ \rho _i(\eta +\omega \,|\,\omega _1,\omega _2)&=\rho _i(\eta \,|\,\omega _1,\omega _2)-a_i\zeta (\eta \,|\,\omega _1,\omega _2)+\sum _{j=1,2}a_j(-a_i\eta _j+(2\eta +\omega )\partial _{\omega _i}\eta _j). \end{aligned}\nonumber \\ \end{aligned}$$

#### Proof

The first and second identity follow from the well-known periodicity of $\wp$ and quasi-periodicity of $\zeta$. On the other hand, the $\sigma$ function satisfies

5.2 $$\begin{aligned} \sigma (\eta +\omega \,|\,\omega _1,\omega _2)=(-1)^{a_1+a_2+a_1a_2}e^{(2\eta +\omega )(a_1\eta _1+a_2\eta _2)}\sigma (\eta \,|\,\omega _1,\omega _2), \end{aligned}$$

from which the last identity follows by taking logs and derivatives with respect to $\omega _i$. Indeed, differentiating the left-hand side of (5.2) gives

5.3 $$\begin{aligned} \partial _{\omega _i}\log (\sigma (\eta +\omega \,|\,\omega _1,\omega _2))=a_i\zeta (\eta +\omega \,|\,\omega _1,\omega _2)+\rho _i(\eta +\omega \,|\,\omega _1,\omega _2), \end{aligned}$$

while differentiating the right hand side gives

5.4 $$\begin{aligned}  &   \partial _{\omega _i}\left( (2\eta +\omega )(a_1\eta _1+a_2\eta _2)+\log (\sigma (\eta \,|\,\omega _1,\omega _2))\right) \nonumber \\  &   \qquad =\rho _i(\eta \,|\,\omega _1,\omega _2)+\sum _{j=1}^2a_j(a_i\eta _j +(2\eta +\omega )\partial _{\omega _i}\eta _j). \end{aligned}$$

The last identity of (5.1) then follows by applying the second identity and reorganizing terms. $\square$

Recall the notion of the irrationality measure $\mu (\alpha )$ of a real number $\alpha \in \mathbb {R}$, and of the irrationality measure $\mu (r)$ of a ray $r\subset \mathbb {C}^*$ with respect to a lattice $\Lambda (\omega _1,\omega _2)$ introduced in Sect. 2.4. In the following, we will use that if $\alpha \in \mathbb {R}{\setminus } \mathbb {Q}$ and $n>\mu (\alpha )$ then

5.5 $$\begin{aligned} |\alpha -p/q|\ge 1/q^n \end{aligned}$$

for all $p,q\in \mathbb {Z}$ with *q* sufficiently large.

#### Lemma 5.2

Fix $(\omega _1,\omega _2)\in \mathcal {R}$ and let $r\subset \mathbb {C}^*$ be a non-Stokes ray such that $\mu (r)<\infty$ with respect to the lattice $\Lambda (\omega _1,\omega _2)$. Then for $\epsilon \in \mathbb {H}_{r}$ the integrals

5.6 $$\begin{aligned} \int _{r} e^{-\eta /\epsilon }\mathcalligra{f}(\eta \,|\,\omega _1,\omega _2)\textrm{d}\eta , \quad \int _{r} e^{-\eta /\epsilon }\mathcalligra{k}_i(\eta \,|\,\omega _1,\omega _2)\textrm{d}\eta , \end{aligned}$$

are absolutely convergent.

#### Proof

The functions $\mathcalligra{f}(\eta )$, $\mathcalligra{k}_i(\eta )$ are meromorphic in $\eta$ with poles only at the nonzero lattice points $\Lambda ^*(\omega _1,\omega _2)$. The fact that *r* is a non-Stokes ray implies that *r* can be parameterised as

5.7 $$\begin{aligned} \eta (t)=\pm t(\omega _1+\alpha \omega _2) , \end{aligned}$$

with $t\in \mathbb {R}_{\ge 0}$ and $\alpha \in \mathbb {R}{\setminus } \mathbb {Q}$. We assume that we are in the case with the $+$ sign, with the other case being completely analogous.

Fix $K>0$. Since the functions $\mathcalligra{f}$, $\mathcalligra{k}_i$ are holomorphic at $0\in \mathbb {C}$, the integrals over $t\in [0,K]$ are finite. Now note that by (2.7)-(2.9), the functions $\tilde{\wp }$, $\tilde{\zeta }$ and $\mathcalligra{k}_i$ differ from $\wp$, $\zeta$ and $\rho _i$ by terms that have at most polynomial growth in $\eta (t)$ as $t\rightarrow \infty$. Due to the exponential decay of $e^{-\eta (t)/\epsilon }$ for $\epsilon \in \mathbb {H}_{r}$ as $t\rightarrow \infty$, to show that (5.6) holds it is then enough to check that

5.8 $$\begin{aligned} \int _{r_{\infty }}|e^{-\eta /\epsilon }\wp (\eta )\textrm{d}\eta |<\infty , \quad \int _{r_{\infty }} |e^{-\eta /\epsilon }\zeta (\eta )\textrm{d}\eta |<\infty , \quad \int _{r_{\infty }} |e^{-\eta /\epsilon }\rho _i(\eta )\textrm{d}\eta |<\infty , \nonumber \\ \end{aligned}$$

where $r_{\infty }$ is the segment given by $\eta (t)$ for $t\in [K,\infty )$. We start with the first of these statements.

We work with the inner product on $\mathbb {C}$ in which $\omega _1$ and $\omega _2$ are orthonormal and consider discs $D_\delta (\omega )$ of radius $0<\delta <K$ centered at the points of $\Lambda (\omega _1,\omega _2)$. We denote the norm induced by this inner product by $|| \cdot ||$ to distinguish it from the canonical norm $| \cdot |$. We take $\delta >0$ sufficiently small so that these discs do not intersect each other. Subdivide the ray $r_{\infty }$ into two sets

5.9 $$\begin{aligned} r_{\infty }=r_p\cup r_c \end{aligned}$$

where $r_p$ is made up of the segments of $r_{\infty }$ inside the discs, and $r_{c}$ is the complement of $r_p$ in $r_{\infty }$. In particular, we can write

5.10 $$\begin{aligned} r_p=\bigcup _{\omega \in \Lambda ^*(\omega _1,\omega _2)}r_{\omega } \end{aligned}$$

where $r_{\omega }$ is the segment contained in the disc $D_\delta (\omega )$.

Take a non-zero lattice point $\omega =a_1\omega _1+a_2\omega _2\in \Lambda ^*(\omega _1,\omega _2)$ and consider

5.11 $$\begin{aligned} \int _{r_{\omega }} |e^{- \eta /\epsilon } \wp (\eta \,|\,\omega _1,\omega _2)\textrm{d}\eta |. \end{aligned}$$

Using the fact that $\wp$ is periodic for the lattice $\Lambda (\omega _1,\omega _2)$ and the Laurent expansion of $\wp (\eta )$ at $\eta =0$ we know that when $\eta \in D_\delta (\omega )$

5.12 $$\begin{aligned} \wp (\eta )=\wp (\eta -\omega )=\frac{1}{(\eta -\omega )^2}+\text {Reg}(\eta -\omega ), \end{aligned}$$

where $\text {Reg}$ is a holomorphic function in the disc $D_\delta (0)$. So in particular we have

5.13 $$\begin{aligned} |\wp (\eta )|=|\wp (\eta -\omega )|\le \frac{1}{|\eta -\omega |^2}+D_1\le \frac{1+C\cdot D_1\delta ^2}{|\eta -\omega |^2}=\frac{D_2}{|\eta -\omega |^2}, \end{aligned}$$

where $D_1>0$, $C>0$ is such that $| \cdot |<C||\cdot ||$, and $D_2=1+C\cdot D_1\delta ^2>0$ are constants independent of $\omega$. Since the canonical norm $|\cdot |$ is equivalent to $||\cdot ||$, it follows that

5.14 $$\begin{aligned} |\eta (t)-\omega |^2\ge D_3((t-a_1)^2 +(t\alpha -a_2)^2) \end{aligned}$$

for some $D_3>0$. Minimizing the right hand side we find

5.15 $$\begin{aligned} (t-a_1)^2 +(t\alpha -a_2)^2\ge \frac{(a_1\alpha -a_2)^2}{1+\alpha ^2}. \end{aligned}$$

By picking $n>\mu (\alpha )$ and possibly increasing $K>0$, we can assume that for all $\omega \in \Lambda ^*(\omega _1,\omega _2)$ such that $r_{\omega }$ is non-empty we have

5.16 $$\begin{aligned} |\alpha -a_2/a_1|\ge \frac{1}{|a_1|^n}, \end{aligned}$$

where we again wrote $\omega =a_1\omega _1+a_2\omega _2$. Hence, overall on $r_{\omega }$ we have

5.17 $$\begin{aligned} |\wp (\eta )|\le \frac{D_2 (1+\alpha ^2)\cdot |a_1|^{2n-2}}{D_3}. \end{aligned}$$

Recall that the discs $D_\delta (\omega )$ are defined with respect to the inner product where $\omega _1$ and $\omega _2$ are orthonormal. The points of intersection of the ray $\eta (t)$ with the boundary of $D_\delta (\omega )$ occur when

5.18 $$\begin{aligned} t=t_{\pm }(\omega )=\frac{(a_1+\alpha a_2)\pm \sqrt{(1+\alpha ^2)\delta ^2-(\alpha a_1-a_2)^2}}{(1+\alpha ^2)}. \end{aligned}$$

Note that if $r_{\omega }$ is not empty, we must have

5.19 $$\begin{aligned} (1+\alpha ^2)\delta ^2-(\alpha a_1-a_2)^2=(1+\alpha ^2)(\delta ^2-\text {dist}(r,\omega )^2)\ge 0, \end{aligned}$$

where $\text {dist}(r,\omega )$ denotes the distance between *r* and $\omega$ in the norm where $\omega _1$ and $\omega _2$ are orthonormal. Hence, we obtain

5.20 $$\begin{aligned} \int _{r_{\omega }}| e^{- \eta /\epsilon } \wp (\eta \,|\,\omega _1,\omega _2)\textrm{d}\eta |\le &   \delta _1|a_1|^{2n-2}\int _{t_{-}}^{t_{+}}e^{-t\delta _2}\textrm{d}t\nonumber \\= &   \delta _1|a_1|^{2n-2}\left( -\frac{1}{\delta }_2(e^{-t_{+}\delta _2}-e^{-t_{-}\delta _2})\right) \end{aligned}$$

where

5.21 $$\begin{aligned} \delta _1=\frac{D_2 (1+\alpha ^2)|\omega _1+\alpha \omega _2|}{D_3}>0, \quad \delta _2 = \left| \frac{\omega _1+\alpha \omega _2}{\epsilon }\right| \cos \left( \text {Arg}\left( \frac{\omega _1+\alpha \omega _2}{\epsilon }\right) \right) >0,\nonumber \\ \end{aligned}$$

so $\delta _1,\delta _2$ are constants depending only on $\alpha ,\omega _1,\omega _2,\delta ,\epsilon$. Furthermore, note that

5.22 $$\begin{aligned} e^{-t_{-}\delta _2}-e^{-t_{+}\delta _2}= &   2e^{-\delta _2\frac{a_1+\alpha a_2}{1+\alpha ^2}}\sinh \left( \delta _2\frac{\sqrt{\delta ^2-\text {dist}(r,\omega )^2}}{(1+\alpha ^2)^{1/2}}\right) \nonumber \\\le &   2e^{-\delta _2\frac{a_1+\alpha a_2}{1+\alpha ^2}}\sinh \left( \frac{\delta _2\delta }{(1+\alpha ^2)^{1/2}}\right) , \end{aligned}$$

so that

5.23 $$\begin{aligned} \int _{r_{\omega }} |e^{- \eta /\epsilon } \wp (\eta \,|\,\omega _1,\omega _2)\textrm{d}\eta |\le 2\frac{\delta _1}{\delta _2}|a_1|^{2n-2}\sinh \left( \frac{\delta _2\delta }{(1+\alpha ^2)^{1/2}}\right) e^{-\delta _2\frac{a_1+\alpha a_2}{1+\alpha ^2}}. \end{aligned}$$

Now note that if $\eta (t)=t(\omega _1+\alpha \omega _2)$ intersects the disc centered at $\omega =a_1\omega _1+a_2\omega _2$, and $\omega$ is sufficiently large in norm, then we must have $a_1,\alpha a_2>0$. In particular, we find that

5.24 $$\begin{aligned} \begin{aligned} \int _{r_{p}} |e^{- \eta /\epsilon }&\wp (\eta \,|\,\omega _1,\omega _2)\textrm{d}\eta |=\sum _{\omega \in \Lambda ^*(\omega _1,\omega _2)}\int _{r_{\omega }} |e^{-\eta /\epsilon } \wp (\eta \,|\,\omega _1,\omega _2)\textrm{d}\eta |\\&<\sum _{(a_1,a_2)\in \mathbb {Z}^2\;:\; r_{a_1\omega _1+a_2\omega _2}\ne \emptyset }2\frac{\delta _1}{\delta _2}|a_1|^{2n-2}\sinh \left( \frac{\delta _2\delta }{(1+\alpha ^2)^{1/2}}\right) e^{-\delta _2\frac{a_1+\alpha a_2}{1+\alpha ^2}}<\infty . \end{aligned} \nonumber \\ \end{aligned}$$

On the other hand, on $r_{c}$ we simply have that due to the periodicity of $\wp$, the factor $\wp (\eta )$ is bounded and hence the integral over $r_c$ is also finite. We then conclude that

5.25 $$\begin{aligned} \int _{r_{\infty }}\left| e^{-\eta /\epsilon }\wp (\eta )\textrm{d}\eta \right| = \int _{r_{c}}\left| e^{-\eta /\epsilon }\wp (\eta )\textrm{d}\eta \right| +\int _{r_{p}}\left| e^{-\eta /\epsilon }\wp (\eta )\textrm{d}\eta \right| <\infty \end{aligned}$$

The argument for the convergence of

5.26 $$\begin{aligned} \int _{r_{\infty }}\left| e^{-\eta /\epsilon }\zeta (\eta )\textrm{d}\eta \right| \end{aligned}$$

is similar. The only difference is that now $\zeta$ is not periodic, so we must use the corresponding identity in Lemma 5.1. This shows that for $\eta (t)\in r_{\omega }$ we have

5.27 $$\begin{aligned} \zeta (\eta )=\zeta (\eta -\omega )+2(a_1\eta _1+a_2\eta _2)=\frac{1}{\eta -\omega }+\text {Reg}(\eta -\omega ) + 2(a_1\eta _1+a_2\eta _2), \nonumber \\ \end{aligned}$$

where Reg as before is a holomorphic function (independent of $\omega$) on a disc of radius $\delta$ centered at 0, so that

5.28 $$\begin{aligned} |\zeta (\eta )|\le \frac{D_1}{|\eta -\omega |}+D_2(|a_1|+|a_2|)<\frac{D_1 (1+\alpha ^2)^{1/2}\cdot |a_1|^{n-1}}{C}+D_2(|a_1|+|a_2|) \nonumber \\ \end{aligned}$$

for some constants $C, D_1,D_2$ independent of $\omega =a_1\omega _1+a_2\omega _2$. The argument for the convergence over $r_p$ follows as before. For the convergence over $r_c$ we again use the quasi-periodicity of $\zeta$ from Lemma 5.1 as before to show that as we go to $\infty$ along $r_c$ we have

5.29 $$\begin{aligned} |\zeta (\eta )|=\mathcal {O}(|\eta |). \end{aligned}$$

Finally, to show

5.30 $$\begin{aligned} \int _{r_{\infty }}\left| e^{-\eta /\epsilon }\rho _i(\eta )\textrm{d}\eta \right| <\infty \end{aligned}$$

we use that

5.31 $$\begin{aligned} \int _{r_{\infty }}\left| e^{-\eta /\epsilon }\zeta (\eta )\textrm{d}\eta \right| <\infty \end{aligned}$$

together with Lemma 5.1 and a simple modification of the argument from before. $\square$

### Proof of the Borel summability

Given the previous lemmas, we now prove the Borel summability of $K_i(\epsilon \,|\,\omega _1,\omega _2)$ and $F(\epsilon \,|\,\omega _1,\omega _2)$.

#### Proposition 5.3

Fix $(\omega _1,\omega _2)\in \mathcal {R}$ and consider a non-Stokes ray *r* such that $\mu (r)<\infty$ with respect to $\Lambda (\omega _1,\omega _2)$. Then for $\epsilon \in \mathbb {H}_r$ the following integrals are absolutely convergent

5.32 $$\begin{aligned}  &   K_{i}^{r}(\epsilon \,|\,\omega _1,\omega _2)=\int _{r} e^{-\eta /\epsilon } k_i(\eta \,|\,\omega _1,\omega _2)\textrm{d}\eta , \quad \nonumber \\  &   F^r(\epsilon \,|\,\omega _1,\omega _2)=\int _{r} e^{-\eta /\epsilon } f(\eta \,|\,\omega _1,\omega _2)\textrm{d}\eta , \end{aligned}$$

and depend holomorphically on $\epsilon$. In particular, the formal series $K_i(\epsilon \,|\,\omega _1,\omega _2)$ and $F(\epsilon \,|\,\omega _1,\omega _2)$ are Borel summable along *r*. Additionally, we have the alternate expressions

5.33 $$\begin{aligned} \begin{aligned} K_{i}^{r}(\epsilon \,|\,\omega _1,\omega _2)&=\int _r \textrm{Li}_1(e^{- \eta /\epsilon })\mathcalligra{k}_i(\eta \,|\,\omega _1,\omega _2)\textrm{d}\eta ,\\ F^r(\epsilon \,|\,\omega _1,\omega _2)&=\int _r \textrm{Li}_2(e^{- \eta /\epsilon })\mathcalligra{f}(\eta \,|\,\omega _1,\omega _2)\textrm{d}\eta . \end{aligned} \end{aligned}$$

#### Proof

Using that along *r* we have $|e^{- \eta /\epsilon }|<1$ and using that

5.34 $$\begin{aligned} |\textrm{Li}_1(z)|=|\log (1-z)|<-\log (1-|z|), \quad |z|<1, \end{aligned}$$

to show the absolute convergence of the first expression of (5.33) it is enough to show the convergence of the integral

5.35 $$\begin{aligned} -\int _r \log (1-|e^{- \eta /\epsilon }|)|\mathcalligra{k}_i(\eta )||\textrm{d}\eta |. \end{aligned}$$

Since near $\eta =0$ we have

5.36 $$\begin{aligned} \mathcalligra{k}_i(\eta )=\mathcal {O}(\eta ) \end{aligned}$$

the integral in (5.35) has no issue near $\eta =0$. On the other hand, as $\eta \rightarrow \infty$ along *r* we have

5.37 $$\begin{aligned} -\log (1-|e^{- \eta /\epsilon }|)\sim |e^{- \eta /\epsilon }|, \end{aligned}$$

so by Lemma 5.2 we have that (5.35) is finite. On the other hand, by Fubini-Tonneli and changing variables we have

5.38 $$\begin{aligned} \begin{aligned} -\int _r \log (1-|e^{- \eta /\epsilon }|)|\mathcalligra{k}_i(\eta )||\textrm{d}\eta |=\,&\int _r \sum _{m\ge 1}\frac{|e^{- m \eta /\epsilon }|}{ m}|\mathcalligra{k}_i(\eta )||\textrm{d}\eta |\\ =\,&\sum _{m\ge 1}\int _r \frac{|e^{-m \eta /\epsilon }|}{m}|\mathcalligra{k}_i(\eta )||\textrm{d}\eta |\\ =\,&\sum _{m\ge 1}\int _r\frac{|e^{- \eta /\epsilon }|}{ m^2}|\mathcalligra{k}_i(\eta /m)||\textrm{d}\eta |\\ =\,&\int _r|e^{- \eta /\epsilon }|\sum _{m\ge 1}\frac{1}{ m^2}|\mathcalligra{k}_i(\eta /m)||\textrm{d}\eta |. \end{aligned} \end{aligned}$$

In the above, we have used that along the ray $|e^{-\eta /\epsilon }|<1$, so that the series expansion of $-\log (1-z)$ is valid along *r*. Since the first integral is finite and $k_i(\eta \,|\,\omega _1,\omega _2)$ is given by (2.18), it follows that the Borel sum $K_{i}^{r}(\epsilon \,|\,\omega _1,\omega _2)$ is absolutely integrable. By applying Fubini-Tonelli to the expressions without absolute values, we also get the alternate identity in (5.33).

The argument for $F^r$ follows similarly. Using that along *r* we have $|e^{- \eta /\epsilon }|<1$ and

5.39 $$\begin{aligned} |\mathrm {Li_2}(z)|\le \mathrm {Li_2}(|z|), \quad |z|<1 \end{aligned}$$

to show the absolute convergence of the second expression in (5.33) it is enough to consider

5.40 $$\begin{aligned} \int _r \textrm{Li}_2(|e^{- \eta /\epsilon }|)|\mathcalligra{f}(\eta )||\textrm{d}\eta |. \end{aligned}$$

Since $\textrm{Li}_2(1)<\infty$ and the modified functions $\widetilde{\zeta }$ and $\widetilde{\wp }$ are finite at $\eta =0$, the integrand does not have any issues at $\eta =0$. Similar to the previous case, as $\eta \rightarrow \infty$ along *r* we have

5.41 $$\begin{aligned} \textrm{Li}_2(|e^{- \eta /\epsilon }|)\sim |e^{- \eta /\epsilon }| \end{aligned}$$

so by Lemma 5.2 we find that (5.40) is finite. By Fubini-Tonneli and performing a change of variables as in (5.38), we find that

5.42 $$\begin{aligned} \begin{aligned} \int _r \textrm{Li}_2(|e^{- \eta /\epsilon }|)|\mathcalligra{f}(\eta )||\textrm{d}\eta |&=\sum _{m\ge 1}\int _r \frac{|e^{-m \eta /\epsilon }|}{m^2}|\mathcalligra{f}(\eta )||\textrm{d}\eta |\\&=\sum _{m\ge 1}\int _r \frac{|e^{- \eta /\epsilon }|}{m^3}|\mathcalligra{f}(\eta /m)||\textrm{d}\eta |\\&=\int _r|e^{- \eta /\epsilon }|\sum _{m\ge 1}\frac{1}{m^3}|\mathcalligra{f}(\eta /m)||\textrm{d}\eta |. \end{aligned} \end{aligned}$$

As before, for the series expansion of $\textrm{Li}_2(z)$ we have used that along *r* we have $|e^{- \eta /\epsilon }|<1$. Since the first integral is finite, and *f* is given by (2.17), it follows that the Borel sum $F^r(\epsilon ,\omega _1,\omega _2)$ is absolutely integrable. By applying Fubini-Tonelli to the corresponding expressions without absolute values, we obtain the alternate identity for $F^r$ in (5.33).

Finally, we show holomorphic dependence in $\epsilon \in \mathbb {H}_r$ for $K^r_i$, with an identical argument for $F^r$. Consider any contour $\partial \Delta \subset \mathbb {H}_r$. Then we clearly have

5.43 $$\begin{aligned} \int _{\partial \Delta }\left( \int _{r}|e^{-\eta /\epsilon }k_i(\eta \,|\,\omega _1,\omega _2)||\textrm{d}\eta |\right) |\textrm{d}\epsilon |<\infty . \end{aligned}$$

By applying Fubini-Tonelli we can interchange the order of integration, and we find

5.44 $$\begin{aligned} \begin{aligned} \int _{\partial \Delta } K_i^r(\epsilon \,|\,\omega _1,\omega _2)\textrm{d}\epsilon&= \int _{\partial \Delta }\left( \int _{r}e^{-\eta /\epsilon }k_i(\eta \,|\,\omega _1,\omega _2)\textrm{d}\eta \right) \textrm{d}\epsilon \\&=\int _{r}\left( \int _{\partial \Delta }e^{-\eta /\epsilon }\textrm{d}\epsilon \right) k_i(\eta \,|\,\omega _1,\omega _2)\textrm{d}\eta =0. \end{aligned} \end{aligned}$$

Hence, by Morera’s theorem it follows that $K_i^r(\epsilon \,|\,\omega _1,\omega _2)$ is holomorphic in $\epsilon \in \mathbb {H}_r$. $\square$

### Integrals along detour paths

When $\mu (\alpha )=\infty$, we can still define something meaningful. The idea is as follows:

- Give a non-Stokes ray *r* with $\mu (r)=\infty$ with respect to the lattice $\Lambda (\omega _1,\omega _2)$, let $r(\delta )$ be the detour path defined in Sect. 2.4 for $\delta$ small enough (see figure 1).
- We then define the following expressions for $\epsilon \in \mathbb {H}_r$
  5.45 $$\begin{aligned} \begin{aligned} K_{i}^{r(\delta )}(\epsilon \,|\,\omega _1,\omega _2)&=\int _{r(\delta )} \textrm{Li}_1(e^{- \eta /\epsilon })\mathcalligra{k}_i(\eta \,|\,\omega _1,\omega _2)\textrm{d}\eta ,\\ F^{r(\delta )}(\epsilon \,|\,\omega _1,\omega _2)&=\int _{r(\delta )} \textrm{Li}_2(e^{- \eta /\epsilon })\mathcalligra{f}(\eta \,|\,\omega _1,\omega _2)\textrm{d}\eta . \end{aligned} \end{aligned}$$
- We will then show the above expressions are independent of $\delta$ for $\delta$ small enough, and they coincide with $K_{i}^{r}$ and $F^r$ when $\mu (r)<\infty$.

#### Proposition 5.4

Let *r* be a non-Stokes ray with respect to $\Lambda (\omega _1,\omega _2)$. Then there is $D>0$ such that for all $0<\delta <D$ the integrals $F^{r(\delta )}$ and $K_{i}^{r(\delta )}$ are absolutely convergent for $\epsilon \in \mathbb {H}_r$. These integrals depend holomorphically on $\epsilon \in \mathbb {H}_r$, and are independent of the choice of such $\delta$. Moreover when $\mu (r)<\infty$ we have $F^{r(\delta )}=F^{r}$ and $K_{i}^{r(\delta )}=K_{i}^{r}$ .

#### Proof

We take $D>0$ such that the discs of radius $0<\delta <D$ and centered at $\Lambda (\omega _1,\omega _2)$ do not intersect each other. Notice that given any parametrization $\eta (t)$ of the corresponding detour path $r(\delta )$ and $\epsilon \in \mathbb {H}_r$, we have $\text {Re}(\eta (t)/\epsilon )>0$ for *t* sufficiently big, so we still have exponential decay as $t\rightarrow \infty$.

On the other hand, the proof of the absolute convergence follows a simpler argument than the one used in Lemma 5.2 and Proposition 5.3. Indeed, one first needs a version of Lemma 5.2 for the detour paths $r(\delta )$. As in Lemma 5.2 we can focus on a segment $r_{\infty }(\delta )$ given by $\eta (t)$ for $t\in [K,\infty )$ and $K>0$ sufficiently big, and furthemore divide $r_{\infty }(\delta )$ into two sets

5.46 $$\begin{aligned} r_{\infty }(\delta )=r_p\cup r_c \end{aligned}$$

where $r_c$ is exactly as in Lemma 5.2, and $r_p$ is now made of the arcs of the detour path, belonging to circles of radius $\delta$ centered at the poles. The argument of the absolute convergence over $r_c$ is exactly the same as in Lemma 5.2, while the estimates for $r_p$ are easier, since we are now always a bounded distance from the poles. For example, when dealing with $\wp (\eta )$, using the periodicity of $\wp (\eta )$ we simply have a uniform bound for $|\wp (\eta )|$ along $r_p$, while for $\zeta (\eta )$ and $\rho _i(\eta )$ we use again Lemma 5.1. One can then apply the same argument of Proposition 5.3 to show that the integrals in (5.45) are absolutely integrable.^2^

Now let $\delta$ be small enough and $0<\delta '<\delta$. Let $r_{n}(\delta )$ (resp. $r_{n}(\delta ')$) be the segment of the detour path $r(\delta )$ (resp. $r(\delta ')$) from 0 to some point between the *n*-th arc and the $(n+1)$-th arc. We pick the endpoint to be the same for $r_{n}(\delta )$ and $r_{n}(\delta ')$ for all $n>0$. Similarly, we denote by $r_n$ the segment of *r* from 0 to the endpoint of $r_{n}(\delta )$. By a trivial argument with contour integrals using the fact that the integrands have poles at $\Lambda ^*(\omega _1,\omega _2)$

5.47 $$\begin{aligned} K_{i}^{r_n(\delta )}=K_{i}^{r_n(\delta ')}=K_{i}^{r_n}, \quad F^{r_n(\delta )}=F^{r_n(\delta ')}=F^{r_n}, \quad \text {for all } n>0 . \end{aligned}$$

When *r* is an arbitrary non-Stokes ray, we can use the existence of $K_{i}^{r(\delta )}$ and $F^{r(\delta )}$ for all small enough $\delta$ and (5.47) to take a limit $n\rightarrow \infty$ and obtain $K_{i}^{r(\delta )}=K_{i}^{r(\delta ')}$, $F^{r(\delta )}=F^{r(\delta ')}$.^3^ Furthermore, when *r* is a non-Stokes ray such that $\mu (r)<\infty$, we can use the existence of $K_{i}^{r}$ and $F^{r}$ and (5.47) to obtain $K_{i}^{r(\delta )}=K_{i}^{r}$, $F^{r(\delta )}=F^{r}$.

Finally, the holomorphicity in $\epsilon \in \mathbb {H}_r$ follows by a the same kind of argument as in Proposition 5.3$\square$

### Stokes jumps

Finally, we discuss how the previous integrals along different paths relate to each other.

#### Proposition 5.5

Let $r_1$ and $r_2$ be two non-Stokes rays ordered in clockwise order, and assume that $\mathbb {H}_{r_1}\cap \mathbb {H}_{r_2}\ne \emptyset$. Furthermore, let $\Delta (r_1,r_2)$ be the sector determined by $r_1$ and $r_2$. Then for $\epsilon \in \mathbb {H}_{r_1}\cap \mathbb {H}_{r_2}$ and small enough $\delta$ we have

5.48 $$\begin{aligned} \begin{aligned} K_{i}^{r_2(\delta )}(\epsilon \,|\,\omega _1,\omega _2)-K_{i}^{r_1(\delta )}(\epsilon \,|\,\omega _1,\omega _2)&=2\pi \textrm{i}\sum _{\omega \in \Delta (r_1,r_2)\cap \Lambda ^*(\omega _1,\omega _2)}a_i\cdot \log (1-e^{-\omega /\epsilon })\\ F^{r_2(\delta )}(\epsilon ,\omega _1,\omega _2)-F^{r_1(\delta )}(\epsilon ,\omega _1,\omega _2)&=2\pi \textrm{i}\sum _{\omega \in \Delta (r_1,r_2)\cap \Lambda ^*(\omega _1,\omega _2)}\frac{\partial }{\partial \epsilon }\left( \epsilon \textrm{Li}_2(e^{-\omega /\epsilon })\right) , \end{aligned}\nonumber \\ \end{aligned}$$

where $$\omega =a_1\omega _1+a_2\omega _2$$.

#### Proof

Consider a sequence $C_n$ with $n>0$ of discs centered at 0 and of radius $R_n$ with $R_n \rightarrow \infty$ as $n\rightarrow \infty$. We denote by $A_n$ the arc of $C_n$ contained in $\Delta (r_1,r_2)$ and assume that $A_n$ does not intersect $\Lambda ^*(\omega _1,\omega _2)$ for all *n*. We orient $A_n$ counter-clockwise. Furthermore, consider $\delta >0$ small enough such that the discs of radius $\delta >0$ centered at the points of $\Lambda (\omega _1,\omega _2)$ do not intersect. We consider detour arcs $A_n(\delta )$, defined similarly to the detour rays $r(\delta )$ by taking a detour along the circles of radius $\delta$ centered at the points of $\Lambda (\omega _1,\omega _2)$ through the shortest length arc. Furthermore, we denote by $\Delta _n(r_1,r_2)$ the region determined by $r_1$, $r_2$ and $A_n(\delta )$, and by $r_{n,1}(\delta )$ and $r_{n,2}(\delta )$ the segments of $r_1(\delta )$ and $r_2(\delta )$ from 0 to the intersection points with $A_n(\delta )$. By using that $\mathcalligra{k}_i(\eta )$ has a simple pole at $\omega =a_1\omega _1+a_2\omega _2\in \Lambda ^*(\omega _1,\omega _2)$ with residue $-a_i$ we obtain using (5.45) that

5.49 $$\begin{aligned} K_{i}^{r_{n,2}(\delta )}-K_{i}^{r_{n,1}(\delta )}+K_{i}^{A_n(\delta )}=2\pi \textrm{i}\sum _{\omega \in \Delta _n(r^1,r^2)\cap \Lambda ^*}a_i\cdot \log (1-e^{-\omega /\epsilon }) \end{aligned}$$

where we have used that $\textrm{Li}_1(z)=-\log (1-z)$. Similarly, using that $\widetilde{\wp }=-\textrm{d}\widetilde{\zeta }/\textrm{d}\eta$ and the definition of $\mathcalligra{f}$, we can use integration by parts on $F^{r(\delta )}$ to write

5.50 $$\begin{aligned} \begin{aligned} F^{r(\delta )}&=\int _{r(\delta )}\left( 2\textrm{Li}_2(e^{-\eta /\epsilon })-\frac{\textrm{d}}{\textrm{d}\eta }(\eta \textrm{Li}_2(e^{-\eta /\epsilon }))\right) \widetilde{\zeta }(\eta ,\omega _1,\omega _2)\textrm{d}\eta \,\\&=\int _{r(\delta )}\left( \textrm{Li}_2(e^{-\eta /\epsilon })+\frac{\eta }{\epsilon }\textrm{Li}_1 (e^{-\eta /\epsilon })\right) \widetilde{\zeta }(\eta ,\omega _1,\omega _2)\textrm{d}\eta \, \end{aligned} \end{aligned}$$

where we have used that the boundary terms of the integration by parts vanish. Using that $\widetilde{\zeta }$ has a simple pole at $\omega \in \Lambda ^*(\omega _1,\omega _2)$ with residue 1 then shows that

5.51 $$\begin{aligned} \begin{aligned} F^{r_{n,2}(\delta )}-F^{r_{n,1}(\delta )}+F^{A_n(\delta )}&=2\pi \textrm{i}\sum _{\omega \in \Delta _n(r^1,r^2)\cap \Lambda ^*}\left( \textrm{Li}_2(e^{-\omega /\epsilon })+\frac{\omega }{\epsilon }\textrm{Li}_1(e^{-\omega /\epsilon })\right) \\&=2\pi \textrm{i}\sum _{\omega \in \Delta _n(r^1,r^2)\cap \Lambda ^*}\partial _{\epsilon }\left( \epsilon \textrm{Li}_2(e^{-\omega /\epsilon })\right) \end{aligned} \end{aligned}$$

Now note that since $\epsilon \in \mathbb {H}_{r^1}\cap \mathbb {H}_{r^2}$ the function $e^{-\eta /\epsilon }$ along $A_n(\delta )$ is exponentially suppressed as $n\rightarrow \infty$. By using a similar argument to Lemma 5.2, Proposition 5.3 and Proposition 5.4 one then finds that

5.52 $$\begin{aligned} \lim _{n\rightarrow \infty }F^{A_n(\delta )}=\lim _{n\rightarrow \infty }K_{i}^{A_n(\delta )}=0\, \end{aligned}$$

and hence the result follows. $\square$

## Data Availability

The authors declare that the data supporting the findings of this study are available within the paper.

## Appendix A. Free Energy in Fibre Classes

Let $\pi :X\rightarrow B$ be an elliptic CY threefold satisfying the assumptions 1.1, and consider the GW generating function in fibre classes of $\pi :X \rightarrow B$ and for genus $g\ge 2$

A.1 $$\begin{aligned} F_{\text {GW}}(\lambda \,|\,Q)=\sum _{g\ge 2}F_g(Q)\lambda ^{2g-2}, \quad F_g(Q)=\sum _{n=0}^{\infty }\text {GW}(g,nF)Q^d. \end{aligned}$$

Here $\text {GW}(g,nF)$ denotes the GW invariant of the class *nF* at genus *g*, and *F* is the fiber class of $\pi :X \rightarrow B$.

   In this Section we show that one can write $F_{\text {GW}}$ as in (1.4). The expression (A.2) below is the same as the one written in [21, Section B.3]. We nevertheless include a more detailed computation for completeness.

### [Style2 Style3 Style3]Proposition A.1

Assuming the GW/DT correspondence holds for *X*, we can write $F_{\text {GW}}(\lambda \,|\,Q)$ from (A.1) as

A.2 $$\begin{aligned} F_{\text {GW}}(\lambda \,|\,Q)=e(X)\sum _{g\ge 2}\frac{(-1)^gB_{2g}}{4g}C_{2g-2}(Q)\lambda ^{2g-2}, \end{aligned}$$

where $C_{2g-2}(Q)$ is the analytic function in *Q* for $|Q|<1$ given by

A.3 $$\begin{aligned} C_{2g-2}(Q)=-\frac{B_{2g-2}}{(2g-2)\cdot (2g-2)!}+\frac{2}{(2g-2)!}\sum _{k,n\ge 1}k^{2g-3}Q^{kn}. \end{aligned}$$

Furthermore, setting $Q=e^{2\pi \textrm{i}\tau }$ for $\text {Im}(\tau )>0$ we have

A.4 $$\begin{aligned} F_{\text {GW}}(\lambda \,|\,\tau )=-e(X)\cdot \sum _{g\ge 2} \frac{B_{2g} \, G_{2g-2}(\tau )}{4g(2g-2)} \left( \frac{\lambda }{2\pi }\right) ^{2g-2}. \end{aligned}$$

### Proof

On the one hand, for $g\ge 2$ and $n=0$ we have the universal contribution of constant maps on a CY threefold *X* [13, Theorem 4]

A.5 $$\begin{aligned} \text {GW}(g,0)=-e(X)\frac{(-1)^gB_{2g}B_{2g-2}}{4g(2g-2)(2g-2)!}\,. \end{aligned}$$

On the other hand, consider the Gopakumar-Vafa form of the Gromov-Witten generating function

A.6 $$\begin{aligned} \sum _{g\ge 0, \; \beta>0}\text {GW}(g,\beta )Q^{\beta }\lambda ^{2g-2}=\sum _{g\ge 0, \; \beta>0, \; k>0}\frac{\text {GV}(g,\beta )}{k}\left( 2\sin \left( \frac{k\lambda }{2}\right) \right) ^{2g-2}Q^{k\beta },\nonumber \\ \end{aligned}$$

where $\text {GV}(g,\beta )$ denotes the Gopakumar-Vafa invariants of the class $\beta$ at genus *g*. Assuming the DT/GW correspondence it is shown in [23, Section 6] that for $\beta =nF$, $n>0$, we have

A.7 $$\begin{aligned} \text {GV}(0,nF)=-e(X), \qquad \text {GV}(1,nF)=e(B), \qquad \text {GV}(g,nF)=0 \quad \text {for} \quad g\ge 2. \nonumber \\ \end{aligned}$$

Hence, restricting to the sum over fiber classes (and denoting $Q^{n\cdot F}=Q^n$ to simplify notation) we find

A.8 $$\begin{aligned} \begin{aligned}&\sum _{g\ge 0, \; n>0}\text {GW}(g,nF)Q^{n}\lambda ^{2g-2}\\&\quad =-e(X)\sum _{k,n>0}\frac{1}{k}\left( 2\sin \left( \frac{k\lambda }{2}\right) \right) ^{-2}Q^{kn}+e(B)\sum _{k,n>0}\frac{Q^{kn}}{k},\\&\quad =e(X)\sum _{k,n>0}\frac{e^{\mathrm {\textrm{i}k\lambda }}}{k(e^{\textrm{i}k\lambda }-1)^2}Q^{kn}-e(B)\sum _{n> 0}\log (1-Q^n). \end{aligned} \end{aligned}$$

Using the generating function of Bernoulli numbers $B_{n}$ and the fact that $B_{n}=0$ for odd $n>1$, one easily finds that

A.9 $$\begin{aligned} \frac{e^{\textrm{i}k\lambda }}{(e^{\mathrm {\textrm{i}k\lambda }}-1)^2}=\sum _{g=0}^{\infty }\frac{(2g-1)(-1)^gB_{2g}(k\lambda )^{2g-2}}{(2g)!}\,, \end{aligned}$$

so when considering only terms with $g\ge 2$ we find that

A.10 $$\begin{aligned} \begin{aligned}&\sum _{g\ge 2, \; n>0}\text {GW}(g,nF)Q^{n}\lambda ^{2g-2}\\&\quad =e(X)\sum _{g\ge 2}\frac{(-1)^gB_{2g}}{(2g)\cdot (2g-2)!}\left( \sum _{k,n>0}k^{2g-3}Q^{k\cdot n}\right) \lambda ^{2g-2}\,\\&\quad =e(X)\sum _{g\ge 2}\frac{(-1)^gB_{2g}}{4g}\left( C_{2g-2}(Q)+\frac{B_{2g-2}}{(2g-2)\cdot (2g-2)!}\right) \lambda ^{2g-2}. \end{aligned} \end{aligned}$$

The expression (A.2) then follows by adding the constant map contribution (A.5).

Finally, to show (A.4) note that the Eisenstein series $G_{2g-2}(\tau )$ has the following expansion for $g\ge 2$, which is a slight rewriting of its Fourier series^4^

A.11 $$\begin{aligned} G_{2g-2}(\tau )=2\zeta (2g-2)\left( 1-\frac{(2\pi )^{2g-2}(-1)^g}{(2g-3)!\zeta (2g-2)}\sum _{k,n>0}k^{2g-3}Q^{kn}\right) , \quad Q=e^{2\pi \textrm{i}\tau }.\nonumber \\ \end{aligned}$$

In the above $\zeta (s)$ denotes the Riemann $\zeta$-function and not the Weierstrass $\zeta$-function that is used in the rest of the paper. From

A.12 $$\begin{aligned} \zeta (2g-2)=\frac{(-1)^g(2\pi )^{2g-2}B_{2g-2}}{2(2g-2)!}, \quad g\ge 2, \end{aligned}$$

it then follows that

A.13 $$\begin{aligned} C_{2g-2}(Q)=-\frac{(-1)^g}{(2g-2)(2\pi )^{2g-2}}G_{2g-2}(\tau )\,. \end{aligned}$$

Hence (A.4) follows from (A.13) and (A.2). $\square$

## Appendix B. Lemma on Irrationality Measure

Recall the definition of irrationality measure $\mu (\alpha )$ from Sect. 2.3. Here we prove that if $\alpha \in \mathbb {R}{\setminus }\mathbb {Q}$ then

B.1 $$\begin{aligned} \mu \bigg (\frac{a\alpha +b}{c\alpha +d}\bigg )=\mu (\alpha )\text { for all } \begin{pmatrix}a& \quad b\\ c& \quad d\end{pmatrix} \in \operatorname {GL}_2(\mathbb {Z}). \end{aligned}$$

It it is enough to show that the result holds for the generators

B.2 $$\begin{aligned} T= \begin{pmatrix}1& \quad 1\\ 0& \quad 1\end{pmatrix}, \quad S= \begin{pmatrix}0& \quad -1\\ 1& \quad 0\end{pmatrix}, \quad P=\begin{pmatrix}1&  \quad 0\\ 0\quad & -1\end{pmatrix}. \end{aligned}$$

of $\textrm{GL}_2(\mathbb {Z})$. This is obvious for the transformations $\alpha \mapsto \alpha +1$ and $\alpha \mapsto -\alpha$ corresponding to *T* and *P*. Thus it remains to prove that $\mu (\alpha )=\mu (1/\alpha )$. By the invariance of $\mu (\alpha )$ under $\alpha \mapsto -\alpha$ we can assume that $\alpha >0$.

Suppose that $0< r<\mu (\alpha )$. By definition of $\mu (\alpha )$, this implies that there are infinitely many $p,q\in \mathbb {Z}$ with $q>0$ such that

B.3 $$\begin{aligned} |\alpha -p/q|<1/q^r. \end{aligned}$$

For a given $q>0$ there can only be finitely many *p* satisfying (B.3). So there must be a sequence $p_n,q_n\in \mathbb {Z}$ with $q_n>0$ satisfying (B.3) such that $q_n\rightarrow \infty$ as $n\rightarrow \infty$. Then

B.4 $$\begin{aligned} \frac{p_n}{q_n}\rightarrow \alpha . \end{aligned}$$

and hence $p_n\rightarrow \infty$ also. Passing to a subsequence we can assume that all $p_n>0$. It follows from (B.3) and (B.4) that for some constant $C>0$

B.5 $$\begin{aligned} \left| \frac{1}{\alpha }-\frac{q_n}{p_n}\right|<\frac{1}{q_n^{r-1}p_n\alpha }=\frac{p_n^{r-1}}{q_n^{r-1}p_n^r\alpha }<\frac{C}{p_n^r}. \end{aligned}$$

If $0<r'<r$ then using the fact that $p_n\rightarrow \infty$ we can assume, after possibly passing to another subsequence, that $p_n^{r-r'}>C$ for all $n\in \mathbb {N}$, and hence that

B.6 $$\begin{aligned} \left| \frac{1}{\alpha }-\frac{q_n}{p_n}\right| <\frac{1}{p_n^{r'}}. \end{aligned}$$

This implies that $r' \le \mu (1/\alpha )$. Since this holds for all $0<r'<r$, it follows that $r\le \mu (1/\alpha )$. But $0<r<\mu (\alpha )$ was chosen arbitrarily so we conclude that $\mu (\alpha )\le \mu (1/\alpha )$. Repeating the argument interchanging $\alpha$ and $1/\alpha$ gives $\mu (\alpha )=\mu (1/\alpha )$.

## Footnotes

1 The multiple sine functions are in turn defined in terms of the multiple gamma functions of Barnes [4].

2 Note that in the case of rays *r* with $\mu (r)=\infty$ we do not know that the expressions (5.33) are absolutely integrable, and hence we cannot directly relate (5.45) to Borel sums by trying to show that $K_{i}^{r(\delta )}$ and $F^{r(\delta )}$ coincide with (5.33). This is because the argument of Proposition 5.3 requires the absolute convergence of (5.33) to show that (5.33) match the corresponding Borel sums.

3 Note that this does not show that $K_{i}^{r(\delta )}=K_{i}^{r}$, $F^{r(\delta )}=F^{r}$, since for a general non-Stokes ray *r* we do not know that $F^r$ and $K_{i}^{r}$ exist, and the existence of a limit along a sequence does not guarantee the existence of the limit.
